# Hippocampus shapes cortical sensory output and novelty coding through a direct feedback circuit

**DOI:** 10.21203/rs.3.rs-3270016/v1

**Published:** 2023-08-23

**Authors:** T. Butola, M. Hernández Frausto, S. Blankvoort, M. S. Flatset, L. Peng, M. Elmaleh, A. Hairston, F. Hussain, C. Clopath, C. Kentros, J. Basu

**Affiliations:** 1 Neuroscience Institute, New York University Langone Health; New York City, 10016, USA.; 2 Department of Neuroscience and Physiology, New York University Grossman School of Medicine; New York City, 10016, USA.; 3 Department of Psychiatry, New York University Grossman School of Medicine; New York City, 10016, USA.; 4 Center for Neural Science, New York University, New York, NY 10003, USA; 5 Centre for Neural Computation, Kavli Institute for Systems Neuroscience, Norwegian University of Science and Technology; Trondheim, Norway.; 6 Institute of Neuroscience, University of Oregon; Eugene, United States.; 7 Department of Bioengineering, Imperial College London; London, SW7 2AZ, UK.

**Keywords:** Plasticity, Electrophysiology, Optogenetics, Rabies virus, Behavior, Memory

## Abstract

To extract behaviorally relevant information from our surroundings, our brains constantly integrate and compare incoming sensory information with those stored as memories. Cortico-hippocampal interactions could mediate such interplay between sensory processing and memory recall^1–4^ but this remains to be demonstrated. Recent work parsing entorhinal cortex-to-hippocampus circuitry show its role in episodic memory formation^5–7^ and spatial navigation^8^. However, the organization and function of the hippocampus-to-cortex back-projection circuit remains uncharted. We combined circuit mapping, physiology and behavior with optogenetic manipulations, and computational modeling to reveal how hippocampal feedback modulates cortical sensory activity and behavioral output. Here we show a new direct hippocampal projection to entorhinal cortex layer 2/3, the very layer that projects multisensory input to the hippocampus. Our finding challenges the canonical cortico-hippocampal circuit model where hippocampal feedback only reaches entorhinal cortex layer 2/3 indirectly via layer 5. This direct hippocampal input integrates with cortical sensory inputs in layer 2/3 neurons to drive their plasticity and spike output, and provides an important novelty signal during behavior for coding objects and their locations. Through the sensory-memory feedback loop, hippocampus can update real-time cortical sensory processing, efficiently and iteratively, thereby imparting the salient context for adaptive learned behaviors with new experiences.

The canonical cortico-hippocampal circuit model posits that hippocampus (HC) receives sensory information from the superficial entorhinal cortex layers 2/3 (EC_L2/3_) for long-term memory representation^9^ but does not send direct hippocampal feedback to it. Instead, hippocampus is believed to project to the deep EC_L5_, which then relays the information back to EC_L2/3_ forming an *indirect* EC_L2/3_-HC-EC_L5_-EC_L2/3_ loop^10^. Here we propose the existence of a *direct* reciprocal EC_L2/3_-HC-EC_L2/3_ loop that bypasses signal transformation and delay incurred by routing of hippocampal signals indirectly via EC_L5_. Such a direct feedback path will allow hippocampus to efficiently modulate cortical information processing and actively shape the cortical sensory information it receives. To understand the circuit architecture and function of the largely unexplored HC-EC feedback, and verify whether in addition to the canonical HC-to-EC_L5_ connection, the hippocampus projects directly to EC_L2/3_ we used anterograde and retrograde viral tracing and functional circuit mapping.

For anterograde tracing, we expressed eYFP in excitatory cells of the output areas of the hippocampus (CA1 and subiculum) by injecting Cre-dependent virus in CamKII-Cre or CamKII-Cre x Ai14 animals (Fig. 1a, Extended Data Fig. 1). We found green eYFP^+^ hippocampal axons not only in EC_L5_ but also in EC_L2/3_ (Fig. 1b–e, Extended Data Fig. 2). We validated the presence of this previously overlooked direct projection from hippocampus to EC_L2/3_ using several viral serotypes expressing various promoters and fluorophores, across a range of injection volumes and viral incubation times. Regardless of strategy, each virus we tested demonstrated the existence of hippocampal projections not only to ECL5 but also to ECL2/3 (Extended Fig. 3a–d). With these experiments we optimized the viral strategy for targeting the hippocampal projections- without aberrant infection in EC. In our analysis, we only included the animals that demonstrated no viral infection in EC cells (Extended Data Fig. 3e–f). Depending on the virus, we found the immunofluorescence intensity of the labeled hippocampal axons in EC_L2/3_ was 2–3 times less than in EC_L5_, suggesting a lower axonal density (Fig. 1c–e). While with most viruses, our injection invariably infected both CA1 and subiculum, the AAV 2.2 known for its restricted spread, allowed us to independently label CA1 and subiculum with two distinct fluorophores. Here we observed that the hippocampal projection axons in EC_L2/3_ predominantly arise from CA1 while subiculum is the main source of canonical HC EC_L5_ projections (Extended Data Fig. 4). Of note is that all the hippocampal projections to EC were limited to medial EC (MEC; Extended Data Fig. 5).

Next, we used retrograde rabies virus tracing^11^ to demonstrate that the principal neurons in MEC_L3_ receive direct monosynaptic inputs from hippocampal CA1. We expressed TVA-2A-rabies G-protein selectively in MEC_L3_ principal neurons (postsynaptic cell) (Fig. 1f) by injecting AAV-tetO-TVAG in layer 3 specific tTA (EC L3 tTA) enhancer mouse line^12^. Using envA pseudotyped deltaG-rRB-GFP (in MEC_L3_) we expressed TVA receptor binding protein with G-deleted rabies virus expressing GFP (Fig. 1g). Combination of G-deleted rabies with G-protein in a subset of postsynaptic cells restricts the retrograde spread of rabies to one upstream neuron (presynaptic cell). Our data (Fig. 1h, Extended Data Fig. 6) demonstrate that indeed MEC_L3_ cells receive direct input from CA1, which comprises 11.9 ± 3.90 % of input contribution. This makes CA1 the third largest input region to MEC_L3,_ following MEC_L5_ (26.7 ± 5.28 %), and pre-post subiculum (22.5 ± 2.12 %), corroborating our anterograde tracing results.

To establish that these anatomically defined HC-EC_L2/3_ projections indeed form functional synapses and drive glutamatergic synaptic input, we investigated their physiological properties by intracellular whole-cell patch-clamp electrophysiology in acute mouse brain slices combined with optogenetic circuit mapping. Using the same anterograde viral injection strategy, we expressed ChR2-eYFP in CA1 pyramidal neurons. First, we photo-stimulated YFP^+^ CA1 pyramidal neuron soma using a 500 ms light pulse to elicit a sustained photocurrent, validating ChR2 infection and function. In the EC, we used a similar protocol to screen for inadvertently infected neurons and excluded any animal with an EC neuron showing a sustained photocurrent (Extended Data Fig. 7). In our hands, the AAV 2.5 serotype is the virus of choice for electrophysiology and behavior experiments, due to its efficiency and efficacy in driving functional opsin expression in hippocampal neurons and their axon terminals, with minimal off-target infection in EC.

Next, we compared the synaptic connectivity and strength of the hippocampal connection to EC_L2/3_ and EC_L5_ layers. For this, we performed intracellular patch-clamp recordings from neurons in both EC layers, while optically stimulating pre-synaptic ChR2^+^ hippocampal projections with focal light pulses (470 nm, 2 ms, Fig. 2a). We observed a higher probability of eliciting light-evoked responses when photostimulating hippocampal projections to EC_L5_ (0.98) compared to EC_L2/3_ (0.88) (Fig. 2b–c). Even at moderate light intensity (10 – 50% LED power), photostimulation of hippocampal axons elicited action potentials (AP) in 28% of the EC_L5_ neurons (Fig. 2b inset). However, even maximum optical stimulation of hippocampal axons failed to elicit spikes in EC_L2/3_ neurons. At maximum light stimulation intensity, we also observed significantly higher peak amplitudes of light-evoked post-synaptic potential (PSP) in EC_L5_ (10.78 ± 0.75; median 11.03) than EC_L2/3_ (4.79 ± 0.71; median 3.94) neurons indicating a stronger HC-to-EC_L5_ than HC-to-EC_L2/3_ synaptic connection (Fig. 2d). Using Tetrodotoxin (TTX; to block Na^+^ channel gated AP firing) and 4- aminopyridine (4-AP; to block voltage gated K^+^ channels and prevent membrane repolarization) we eliminated polysynaptic responses^13^ and showed a higher probability of monosynaptic connection between the HC-EC_L5_ (0.82) than HC-EC_L2/3_ (0.41) (Fig. 2e). Since the hippocampal projections are capable of driving EC_L5_ neurons to fire, it is likely these contribute to some of the polysynaptic light-evoked responses we recorded in EC_L2/3_.

Could the smaller net depolarization driven by the HC-EC_L2/3_ inputs compared to their HC-EC_L5_ counterparts be due to a difference in the excitation and inhibition balance exerted by each pathway? To test this, we performed voltage-clamp recordings at −80 mV and +10 mV to directly isolate the light evoked excitatory and inhibitory post synaptic currents (EPSC and IPSC respectively) in L5 and L2/3 pyramidal neurons upon photostimulation of HC inputs (Fig. 2f). We found that EC_L5_ neurons (1.69 nA ± 0.27; median 1.61 nA) receive comparable inhibition as EC_L2/3_ neurons (1.64 nA ± 0.32; median 1.43 nA). However, the excitation at EC_L5_ neurons (−1.00 nA ± 0.17; median −0.83 nA) is much greater than at EC_L2/3_ neurons (−0.43 nA ± 0.10; median −0.27 nA; Fig. 2g, Extended Data Fig. 8). As a result, the excitation to inhibition ratio is significantly lower at the HC EC_L2/3_ (0.25 ± 0.03; median 0.24) synapse than at HC EC_L5_ synapse (0.67 ± 0.13; median 0.55; Fig. 1h). Overall, hippocampal inputs recruit strong excitation upon EC_L5_ neurons, but predominantly feed-forward inhibition upon EC_L2/3_ neurons.

Next, to explore how the hippocampal feedback shape cortical sensory processing, we tested whether hippocampal inputs gain-modulate the sub- and supra-threshold input-output transformation of the multimodal sensory inputs arriving at EC layer 1 (L1)^10,14^ at the distal dendritic compartment of EC_L2/3_ vs. EC_L5_ neurons. Specifically, we tested if repetitive coupling of photostimulation of hippocampal input with electrical stimulation of the L1 sensory input (Fig. 3a–b, L1 before HC, 20 ms apart repeated 90x at 1 Hz) could induce input timing dependent plasticity (ITDP^15–17^) in EC neurons. Typically, given its distal dendritic location, the responses evoked by EC_L1_ input stimulation in both EC _L2/3_ and EC_L5_ are small in amplitude (6.22 2.44 mV (median 4.34 mV) at ECL_2/3_, 4.05 1.15 mV (median 4.02 mV) at ECL_5_) and rarely reach spike threshold (0% spike probability). In EC_L2/3_ neurons, L1-HC pairing induced robust long-term potentiation (LTP) of the L1 sensory evoked response by 1.75 0.24 fold (median 1.4 fold), without a change in the hippocampal input driven light evoked responses (0.93 0.09 fold median 0.95 fold). Moreover, within minutes of L1-HC pairing induction, the L1 stimulation alone started eliciting APs in EC_L2/3_, reaching a striking spike probability of 55.15 10.44 % (median 62.69 %) (Fig. 3c). In contrast, the same pairing paradigm in EC_L5_ neurons only slightly potentiated L1 evoked sub-threshold responses (1.12 0.03 fold; median 1.11 fold) with no impact on spike probability (0% pre and post induction). Whereas the hippocampal input driven light evoked responses at EC_L5_ neurons potentiated significantly (1.40 0.08 fold; median 1.38 fold, Fig. 3d). Thus, the weaker and sparser HC-EC_L2/3_ input is capable of heterosynaptically modulating the sensory output in the EC through induction of long-term ITDP and enhancing pathway specific excitability. Pairing the stronger and denser HC-EC_L5_ with L1 sensory inputs plays only a minor role in modulation of cortical sensory output and mainly induces homosynaptic plasticity of its own output.

The direct projections from hippocampus to (EC_L2/3_), the *very* layers that provide it multisensory input, confirms the existence of a true EC_L2/3_ HC EC_L2/3_ reciprocal feedback loop. We postulated whether reciprocally driven activity in this cortico-hippocampal feedback circuit could iteratively refine cortical output and improve learning performance similar to how feedback loops improve artificial neural network performance in machine learning^18–20^. To test this hypothesis, we built a rate-based circuit model consisting of two sensory inputs (S_1_ and S_2_) projecting with the weights, w_1_ and w_2_ onto the read-out neuron in EC_L2/3_ (Fig. 3e). We simulated the ITDP observed in our slice experiment (Fig. 3a–d) by pairing HC-EC_L2/3_ reciprocal feedback with S_1_. We first recapitulated the acute slice condition in Case 1 (Fig. 3f) where HC-EC_L2/3_ feedback was intact but EC_L2/3_-HC connections were severed due to plane of sectioning. Pairing hippocampal feedback with S_1_ sensory input leads to potentiation of S_1_, increasing the weight w_1_ and the readout activity (as a proxy to behavioral output) 1.5 folds, similar to our experimental ITDP data. As expected, the output of the unpaired sensory input (S_2_) remains unchanged. This suggests that the HC-EC_L2/3_ input is sufficient for inducing potentiation in the model. We also established the requirement of the HC-EC_L2/3_ direct input as silencing HC-EC_L2/3_ feedback abolished LTP (data not shown). Next to answer whether learning rate is increased with reciprocally driven activity between cortex and hippocampus, we simulated (in Case 2, Fig. 3g) the *in vivo* condition where the whole EC_L2/3_ HC EC_L2/3_ loop is intact. In this condition, the same number of pairings of S_1_ with HC-EC_L2/3_ input and the subsequent reciprocal activation of the feedback circuit loop sped up the learning rate and lead to a higher potentiation of S_1_ pathway, with a 2.29-fold boost in input weight w_1_ and in the behavioral output. Thus, our computational model suggests that reverberating activity in the reciprocal connections between hippocampus and EC_L2/3_ leads to a true feedback circuit loop which iteratively improves the learning and output behavior of the system.

What is the *in vivo* functional role of this direct but weaker and sparser hippocampal feedback circuit to EC_L2/3_? For this, we used a loss of function approach to optogenetically silence the HC-EC_L2/3_ circuit during freely moving spatial, contextual, and spatio-contextual memory tasks (Fig. 4). We virally expressed (i) far red light gated inhibitory opsin JAWs (Test cohort) (Extended Data Fig. 9a–c) in glutamatergic hippocampal neurons in dorsal CA1 and (ii) implanted fiber-optic cannulae over EC_L2/3_ (Fig. 4a–b). This way, we could locally inhibit the HC-EC_L2/3_ circuit with temporal precision, during the recall phases of these behaviors. We similarly photostimulated control animals that expressed EGFP instead of JAWs. The hippocampal feedback projections were restricted to the MEC (Extended Data Fig. 5), the hub of spatial information processing^21,22^. Therefore, we first tested the effect of HC-EC_L2/3_ circuit silencing in animals trained to solve the Barnes maze^23^, a classical spatial memory task. Here animals were trained to find an escape hole over a course of 7 days. During the memory recall phase, on day 8, we photostimulated all animals and quantified the number of errors made and the time taken to find the escape hole (Fig. 4c). Animals in both cohorts reliably learnt the task over 7 days as demonstrated by the significantly low error rate (Extended Data Fig 9d) and latency (Extended Data Fig 9e) on day 7 compared to the day 1. Silencing the HC-EC_L2/3_ circuit had no significant impairment in the animal’s performance during spatial memory recall in the Barnes maze task (Fig. 4d–e).

Human and animal studies propose the role of hippocampal area CA1 as a match-mismatch detector for comparing past and present experience driven by predictive and novelty inputs^24–29^. Thus, we tested whether activation of HC-EC_L2/3_ circuit during memory retrieval is necessary for novelty detection using novel object recognition^30,31^ (NOR) (Fig. 4f) and novel object location^32,33^ (NOL, Fig. 4g) tasks. In both these tasks, the animals were habituated to an empty arena for two days prior to object exposure (10 mins/day). After this habituation phase, on Day 3, the animals explored two identical objects placed in the arena in the NOR task (10 mins/ session encoding phase). We found that both control and JAWS animals explored the two objects for comparable time (Extended Data Fig. 9f). Following a 30-minute wait period, which typically engages hippocampal dependent memory formation, the animals were returned to the arena with one familiar and one novel object. We tested for memory recall by comparing the time spent by the animals exploring each object (10 mins, recall test phase). Both cohorts were photostimulated during this test phase. While there was no difference in the time spent exploring the familiar object by the two cohorts (Extended Data Fig. 9g), control animals expectantly explored the novel object for a significantly longer time (37.94 ± 8.88 s; median 29.39 s) compared to the familiar object (26.51 ± 5.93 s; median 20.70 s). In contrast, animals where HC-EC_L2/3_ circuit was silenced spent significantly less time exploring the novel object (14.99 ± 5.17 s; median 7.54 s) compared to the familiar one (20.54 ± 6.11 s; median 12.30 s) (Fig. 4h). As a result, the JAWs cohort demonstrated a significantly reduced NOR index (0.35 ± 0.04 (median 0.33) JAWs vs 0.60 ± 0.02 (median 0.61) control; Fig. 4i) indicating an impairment in the animals’ ability to discriminate between novel vs. familiar objects. Additionally, the control cohort demonstrated a positive discrimination index (0.20 ± 0.03; median 0.21) indicating a preference for the novel object. The JAWS cohort showed the opposite effect, with a significantly different negative discrimination index (−0.30 ± 0.08; median −0.34) indicating a preference for the familiar object (Extended Data Fig. 9h).

Finally, to test whether spatio-contextual associative novelty depends on the HC-EC_L2/3_ circuit, we performed NOL task on Day 4, where the animals were allowed to explore two identical objects placed at specific locations in the arena. We found that the animals spent comparable length of time at each object location (Extended Data Fig. 9i). In the test phase, we displaced one of the objects to another location in the arena, while the other remained in its original location. The two cohorts spent comparable time exploring the familiar object locations (Extended Data Fig. 9j). Control animals expectantly explored the novel object location for a significantly longer time (37.62 ± 6.10 s; median 31.02 s) compared to the familiar object location (26.86 ± 5.45 s; median 22.40 s). In contrast, animals where HC-EC_L2/3_ circuit was silenced spent comparable time with both the novel object location (20.49 ± 4.81 s; median 20.31s) and the familiar one (29.50 ± 9.41 s; median 22.58 s) (Fig. 4j). As a result, the JAWs cohort demonstrated significantly reduced NOL index (0.49 ± 0.05 (median 0.49) JAWs vs 0.60 ± 0.01 (median 0.58) control; Fig. 4k) indicating an impairment in the animals’ ability to recognize novel vs. familiar locations of the object. The control cohort demonstrated a positive discrimination index (0.19 ± 0.03; median 0.16) indicating a preference for the novel object location. In contrast, JAWS cohort showed a near-zero discrimination index (−0.01 ± 0.01; median −0.01) indicating no preference for any of the locations. (Extended Data Fig. 9k). Our data suggest that at the behavioral level, *in vivo* silencing of the HC-to-EC_L2/3_ circuit impairs novelty detection in object recognition and object-place association tasks.

In summary, our study set out to explore whether the back-projection circuit from hippocampus to entorhinal cortex may serve as the circuit substrate for hippocampus to exercise modulation of cortical sensory processing. Our findings show that hippocampus not only projects to the deep layers of EC but also directly to the superficial layers, from which it receives multimodal sensory input. We provide the first anatomical, physiological and behavioral analysis of the significance of this direct hippocampal feedback in modulation of cortical sensory processing. Our study suggests that this direct pathway may act as an “expressway” for hippocampal feedback to shape sensory processing in the entorhinal cortex, bypassing any signal lag and input transformations imposed by the canonical indirect circuit organization. Importantly, our study also provides the first functional mapping and characterization of the canonical HC-EC_L5_ circuit.

Although once described anatomically in rats^34^, most rodent studies^10,35^ have overlooked existence of HC-EC_L2/3_ feedback projections. This could be due to species specific differences or limitations of traditional tracing methods which provided the basis of the canonical circuit model. Our study used the latest anterograde and retrograde tracing approaches optimized for high resolution and cell type specific circuit mapping. This enabled us to resolve even the hidden sparse direct hippocampal projection axons to EC_L2/3_ distinct from the prominent HC-EC_L5_ projections. While CA1 and especially, CA2 have been shown to directly project to EC_L2_^11^, the projection from CA1 to EC_L3_ seems to be more significant. Inputs to EC_L2_ are dominated by pre/parasubicular and intraentorhinal inputs (combined 77.69 %, as opposed to 2.76 % and 11.96 % from CA1 and CA2 respectively), while EC_L3_ receives its third largest input from CA1, after only EC deep layers and presubiculum.

The two parallel processing pathways (HC-EC_L2/3_ and HC-EC_L5_) have key physiological differences that exert distinct synaptic transformations. Hippocampal inputs recruit strong excitation upon EC_L5_ neurons, but predominantly feed-forward inhibition upon EC_L2/3_ neurons. Similar to the auditory system^36^, the small depolarizing potentials evoked by basal activity in HC-EC_L2/3_ synaptic input may provide the graded changes that are needed for the fine scale adjustment in signal modulation. In contrast, such fine range tuning cannot be achieved by the large PSPs (at times all or nothing APs) generated by the strong HC-EC_L5_ input. Thus, despite being sparser and weaker, the synaptic inputs from HC-EC_L2/3_ modulate the sensory input-output gain of EC_L2/3_ neurons by potentiating paired sub-threshold sensory input evoked responses through heterosynaptic plasticity mechanisms and boosting their probability of driving supra-linear output. In contrast, identical pairing of the HC-EC_L5_ inputs with the sensory inputs results in homosynaptic potentiation of its own output, and may be involved in rate coding functions.

The differences in signal transformation observed in the two pathways could reflect differences in circuit architecture and dendritic summation of the inputs upon the EC neurons. In the hippocampus, the spatio-temporal learning rule in ITDP derives from the delay-line architecture of direct and indirect EC inputs onto CA1 PNs. The precisely timed EC and CA3 input stimulation induces ITDP, which results in heterosynaptic potentiation of the CA3-CA1 synapse and depression of local inhibition^15,16^. Similar convergence likely exists in EC: inputs from sensory cortices arrive in EC_L1_^10^, and inputs from CA1 arrive in EC_L2/3_ and EC_L5_ converging onto distinct dendritic compartments of EC neurons^10,37^. Both EC_L2/3_ and EC_L5_ would receive hippocampal inputs along their proximal dendrites, which might intercept sensory input (arriving in EC_L1_) propagating from the distal dendrites. Thus, the inputs converging onto EC dendrites likely also give rise to a delay-line architecture, and allow for precise spatiotemporal coupling of sensory and hippocampal inputs. Since the converging inputs would have different distances to travel along the dendrites of EC_L2/3_ and EC_L5_ neurons, the time windows for the various signal integrations necessary for ITDP to occur may differ in the two EC layers. Future studies are needed to explore the various time windows for the various signal integrations necessary for ITDP to occur in EC_L5_ just as seen in EC_L2/3_ with a 20 ms window of hippocampal and sensory input pairing. With modeling, we showed that iterative feedback from the HC-EC_L2/3_ speeds up learning and that both learning and plasticity rely on the HC-EC_L2/3_ pathway activity. Our findings suggest that hippocampus and entorhinal cortex form a reciprocal feedback loop for integration of mnemonic and multisensory information, where direct input from hippocampus modulates cortical sensory processing by iteratively potentiating associative EC output.

Excitatory drive from hippocampus has been postulated to be important for supporting grid cell activity in MEC^38^. Given that grid cells are primarily in MEC_L2/3_^39^ and HC-EC_L2/3_ feedback inputs are concentrated in MEC (our study), we expected that silencing of this circuit would impact spatial memory guided behavior. Surprisingly, we found that the HC-EC_L2/3_ feedback circuit is not required for recall of spatial memory, but rather discrimination of object or place related novelty tasks traditionally attributed to contextual coding functions of hippocampus and lateral EC (LEC)^40,41^. A possible explanation for this is that the hippocampal feedback circuit enables the cortex to be privy of contextually rich mnemonic associations. Hippocampal CA1 integrates multimodal sensory inputs containing *both* spatial and contextual information from MEC and LEC, respectively. Further, in service of its match-mismatch detection function^24–29^, CA1 compares the online sensory information (*arriving from MEC and LEC)* with predictive memory representations processed intra-hippocampally (*through the EC DG CA3 CA1 trisynaptic pathway)*. We propose that hippocampus drives the instructive signal for cortex to learn about differences in an object’s identity or location. During memory recall, the direct HC-EC_L2/3_ feedback loop iteratively boosts the salience of associative sensory signals, imparting environmental representations with the perception of novelty and familiarity.

Our study provides a first description of a circuit that might form a substrate for integration of sensory processing with memory in the healthy brain. Inappropriate integration of memory and sensory inputs stemming from abnormalities in this HC-EC circuit may underlie the memory-related sensory processing deficits observed in autism, schizophrenia, post-traumatic stress disorder, and Alzheimer’s disease. Thus, future studies need to investigate the pathological modifications in the structure and function of the reciprocal HC-EC feedback loop. The architecture and functional impact of this novel input warrant an updated view of the reciprocal cortico-hippocampal circuit model, whereby hippocampal feedback can now actively shape the cortical sensory information it receives.

## Methods

### Animals

All experiments were conducted in accordance with the National Institutes of Health guidelines and with the approval of the *New York University School of Medicine Institutional Animal Care and Use Committee (IACUC).* Mouse lines were obtained from the Jackson Laboratory (JAX) and subsequent breeding was established in house. CamKII Cre^42^ (The Jackson Laboratory; Jax stock #005359) mice were used to target excitatory neurons. In a subset of experiments to visualize excitatory neurons, CamKII Cre mice were crossed with homozygous Cre-dependent Ai14-tdTomato reporter mice^43^ (The Jackson Laboratory; Jax stock #007914) to generate homozygous CamKII Cre x Ai14 mice. For retrograde rabies virus tracing, we used EC-L3-tTA transgenic mice described as Dok5-LEC-13–8A in Blankvoort et al. 2018^12^. This line expresses a transgene almost exclusively in entorhinal cortex layer 3 (EC_L3_) neurons, with minimal expression in EC_L2_. To ensure that these results were not due to a peculiarity of the enhancer line, we performed an injection set on a second EC_L3_-specific line which used a different enhancer, which gave similar results. For clarity’s sake, throughout this publication we refer to both these lines as the “EC-L3-tTA” line. These experiments were approved by the local ethics committee and in accordance with the European Communities Council Directive and the Norwegian Experiments on Animals Act.

Adult mice of either sex were used for the various experiments. Age of the mice used is specified in the subsection pertaining to the experiments. Litter mates were co-housed up to 5 mice per cage and had *ad libitum* access to food and water.

### Viruses

For anterograde viral tracing and optogenetic activation of hippocampal projections to entorhinal cortex in electrophysiology experiments, AAV 2.5-EF1a-double floxed-hChR2 (H134R)-EYFP-WPRE-HGHpA (a gift from Karl Deisseroth; Addgene viral prep # 20298-AAV5; http://n2t.net/addgene:20298; RRID: Addgene_20298) was injected unilaterally (7 sites, 23 nl injected per site unless otherwise stated in figure legends) into dorsal hippocampal area CA1 of mice under stereotactic control. Acute slice experiments were conducted 21 – 23 days after the injection. After electrophysiology experiments, the same slices were fixed and used for immunohistochemistry experiments for anterograde viral tracing. The virus serotype, volume of injection per site and the duration of incubation were very carefully titrated and optimized to avoid infecting the downstream synapses in the EC.

In the pilot phase we tested three other viral serotypes: AAV 2.1^44^ (Addgene_20298), AAV 2.2 (pAAV-CaMKIIa-hChR2 (H134R)-EYFP was a gift from Karl Deisseroth (Viral packaging Penn Vector core and Addgene viral prep # 26969 ; http://n2t.net/addgene:26969; RRID:Addgene_26969), and AAV 2.9^44^ (Addgene_20298). Although we observed a stronger fluorescence intensity in both EC_L2/3_ and EC_L5_ using these serotypes, the rate of aberrant EC infection in serotypes AAV 2.1 and AAV 2.9 was much higher (Extended Fig. 3). Similar to AAV 2.5, for AAV 2.2 we did not observe infected EC neurons up to 3 weeks post injection. However, with AAV 2.2 we were unable to elicit any light evoked responses upon optical stimulation of 470 nm used to activate ChR2.

To resolve the HC-EC_L5_ and HC-EC_L2/3_ feedback projections, in a subset of experiments we used AAV 2.2-Syn-FLEX-rc [Chronos-GFP]^45^ (a gift from Edward Boyden; Addgene viral prep # 62722; http://n2t.net/addgene:62722; RRID:Addgene_62722) and AAV 2.2-Syn-FLEX-rc[ChrimsonR-tdTomato]^45^ (a gift from Edward Boyden; University of North Carolina (UNC) viral prep, Addgene viral prep # 62723; http://n2t.net/addgene:62723 ; RRID:Addgene_62723). We injected 92 nl/site specifically targeting CA1 and subiculum, and alternated the two viruses between the two brain regions. For retrograde rabies virus tracing, we used a combination of helper virus containing TVA and rabies G, AAV 2.1-tetO-TVA-HA-csvG prepared as described earlier^46^ (referred here as “AAV-tetO-TVA-2A-G”) and an EnvA pseudotyped, SAD-B19 strain prepared as described earlier^47^ (referred here as “ΔG-rRB-GFP”). This allows for specific (only tTA expressing cells) entry of an envA pseudotyped G-deleted (ΔG) rabies virus carrying GFP (“ΔG-rRB-GFP”), and subsequent mono-synaptic retrograde transport due to the rabies-G supplementation in the transgene expressing cells. To limit the starter cells verifiably to the MEC, we injected a helper virus containing TVA and rabies G (“AAV-tetO-TVA-2A-G”) 2 – 3 weeks before ΔG-rRB-GFP.

For *in vivo* optogenetic silencing in freely moving animal behavior experiments, we injected AAV 2.5-CAG-FLEX-rc [Jaws-KGC-GFP-ER2]^48^ (a gift from Edward Boyden; Addgene viral prep # 84445-AAV5; http://n2t.net/addgene:84445; RRID: Addgene_84445) in test JAWs cohort and AAV 2.5-CAG-FLEX-EGFP-WPRE^49^ in the control cohort (a gift from Hongkui Zeng; Addgene viral prep # 51502; http://n2t.net/addgene:51502; RRID:Addgene_51502) bilaterally into dorsal CA1 of mice. The JAWS virus was incubated for 15 – 18 days until we observed a functional expression in our acute slice electrophysiology experiments. The behavior was setup in such a way that the ‘test trials’ with optical JAWS silencing were performed at least 12 – 15 days post injection to give JAWS sufficient incubation time for reliable expression.

### Stereotactic surgery

#### For expressing ChR2, JAWS, and EGFP in hippocampal neurons

Viral injection pipettes were prepared using thin glass pipette (1.14 mm O.D. × 3.5″ length × .53 mm I.D., Drummond Scientific # 3–000-203-G/X), pulled by a micropipette puller (Sutter P-1000, Sutter Instrument Company) and fire polished using a microforge (Narishige Co. Ltd., Tokyo, Japan) to have a long taper and 7–10 μm tip diameter. Pipettes were back-filled with mineral oil, then front-filled with the virus (AAV 2.5-EF1a-double floxed-hChR2 (H134R)-EYFP-WPRE-HGHpA or AAV 2.5-CAG-FLEX-rc [Jaws-KGC-GFP-ER2] or AAV 2.5-CAG-FLEX-EGFP-WPRE) using a Nanoject II injector (Drummond Scientific). Adult mice (6–7 weeks old for ChR2 and 8–16 weeks old for JAWS and EGFP) were injected using stereotactic surgery and sterile technique. The animal was deeply anesthetized with inhalation anesthetic – isoflurane (Covertus, SKU #029405; 5% for induction, 1.5–2% for maintenance during surgery, Matrx VIP 3000 calibrated vaporizer), and was given an i.p. injection of 0.05–0.1 mg/kg buprenorphine HCl (0.3mg/ml, Covertus) before surgery. After head-fixation of the animal in a stereotactic frame (Stoelting), eye ointment was applied and the surgical area was prepared with aseptic technique. The hair on the mouse’s head was shaved with a razor, and the exposed skin was locally disinfected with ethanol and betadine. Before incision, we topically applied ropivacaine HCl (1–3 drops; 2mg/ml, Hospira) to the exposed skin as a local analgesic. An incision was made to expose the skull, and hydrogen peroxide (0.1%) was applied to the surface of the skull to clean it. The skull was levelled such that Bregma and Lambda were aligned along the z-axis.

A craniotomy was made by thinning the skull with a dental drill (Foredom Electric Co.). A skull cap large enough to encompass the injection coordinates was removed leaving the pia intact. After gaining access to the surface of the brain, the injection pipette was lowered onto the pia. A dimple forms on the pia upon contact with the injection pipette. The pipette was retracted enough to eliminate the dimple, and the z – coordinate was zeroed on the flat pial surface. Then the injection pipette was advanced in to the brain to target the dorsal CA1. The loci of the injection sites were as follows:

**Table:** 

**medial-lateral relative to midline (x)**	−1.40	−1.50	−1.70
**anterior-posterior relative to bregma (y)**	−2.00	−2.20	−2.40
**dorsal-ventral relative to surface of the brain (z)**	−1.00	−1.10	−1.10
	−1.10	−1.25	−1.30
*all co-ordinates in mm.*	−1.20		

The injection site coordinates to target subiculum and CA1 in the same mouse brain were as follows:

**Table:** 

**Brain Region**	Subiculum	CA1
**medial-lateral relative to midline (x)**	−0.50	−1.50
**anterior-posterior relative to bregma (y)**	−2.30	−2.30
**dorsal-ventral relative to surface of the brain (z)**	−1.40	−1.10
*all co-ordinates in mm.*		

The pipette was lowered into the brain, and the virus was injected into each coordinate (23 nl injected per site unless stated otherwise in the figure legend). After the final coordinate in the injection site, the pipette was left in place for 10–15 minutes to allow for adequate dispersion of the virus. After slowly withdrawing the injection pipette from the brain, the incision was sutured (Henry Schein) and antibiotic ointment (Neosporin) was applied (in case of JAWS injection, the animals proceeded for optical fiber implantation, see below). Animals were then injected with 0.5–1.0 ml saline solution to rehydrate. The animals were given an i.p. injection of 0.05–0.1 mg/kg buprenorphine per day for three days following the surgery.

We carefully optimized the injection volume and the incubation period to restrict the viral infection to the site of injection – dorsal CA1. For quantitative analysis of fluorescence and physiological characterization of hippocampal feedback projections to EC we carefully screened for aberrant viral infection, and discarded all data from any animal that exhibited infected neurons in the EC. However, due to the extended experimental time required to execute the behavioral tasks we observed some infected (eYFP^+^) neurons in the EC_L2/3_ (no infected neurons were observed in EC_L5_). Thus, instead of silencing exclusively the hippocampal projections to EC_L2/3_ in our behavioral assay, it is likely we silenced at least in part the EC_L2/3_ HC EC_L2/3_ loop in effect.

#### For optical fiber implantation

To silence JAWS^+^ hippocampal projections and EC_L2/3_ neurons, red light (625 nm) was delivered through 200 μm optic fiber (Thorlabs, FT200EMT) coupled to a ceramic ferrule (Thorlabs, CFLC230) implanted 50 μm above EC_L2/3_. To obtain a smooth and clean tip for optimal light transmission, the optical fibers were polished by sanding the tip using fiber polishing film of decreasing grit size (Thorlabs, LF30D (30 μm) > LF6D (6 μm) > LF3D (3 μm) > LF1D (1 μm) > LFCF (0.02 μm), grit size in parentheses). The optical fiber tip quality was checked using a fiber inspection scope (Thorlabs, FS201). We only used the optic fibers that had a smooth clean tip surface and transmitted a smooth circle of light onto a black surface with at least 75% of original laser intensity (25% of leaking, measured using optical power meter (Thorlabs, PM100D)). The fibers were implanted bilaterally in the mouse brain following the bilateral injection of AAV 2.5-CAG-FLEX-rc [Jaws-KGC-GFP-ER2] and AAV 2.5-CAG-FLEX-EGFP-WPRE into dorsal CA1 of 8–16 weeks old mice.

After injecting the virus into the mouse brain we i.p. injected the animals with 0.5–1.0 ml saline solution to rehydrate. We then switched the injection pipette with the optical fiber assembly to be implanted. We used a straight clamp with pad (Kopf, 1271-C-MOD) to hold the optical fiber assembly onto the electrode holder used during the virus injection. We then changed the angle of approach of the fiber holder to 8°. We then zeroed the x – coordinate on to the true lambda (instead of the stereotaxic lambda) suture. We then moved to the desired x – coordinate for the implant site (x = 3.85). At this x – coordinate we located the transverse sinus posterior to the lambda suture. We cleared the tissue over sinus, faintly visible through the skull, with forceps and 0.9 % sterile saline solution. We zeroed the y – coordinate at the most anterior edge of the sinus. We drilled a hole through the skull at x = 3.85 and y = −0.30 (anterior to the sinus). The optical implant was lowered into the brain 1.00 mm from the pial surface. The implant was allowed to rest for 1 minute before retracting and reinserting to be secured to the mouse brain using dental cement (Parkell). After the implantation, the animals were again injected with 0.5–1.0 ml saline solution to rehydrate. The animals were given an i.p. injection of 0.05–0.1 mg/kg buprenorphine per day for three days following the surgery. After eight days of virus incubation period the animals were taken through a battery of behavioral tasks.

#### For Rabies virus tracing

Animals were anesthetized with isoflurane in an induction chamber (5%, Nycomed Zurich, airflow 1.2 l/min). The top of their scalp was shaved with an electrical shaver, eye ointment was applied and animals were injected subcutaneously with general analgesics buprenorphine hydrochloride (0.1 mg/kg, Temgesic, Indivior), meloxicam (1mg/kg, Metacam, Boehringer Ingelheim Vetmedica) and near the incision site with local analgesic bupivacaine hydrochlorine (1mg/kg, Marcain, AstraZeneca). After this, the animal head was fixated in a stereotactic frame (Kopf Instruments, Tujanga, CA) with earbars and anesthesia was maintained with isoflurane (1–1.5%, airflow 1.2L/min). The scalp was disinfected with chlorhexidine and 70% ethanol, after which a rostro-caudal incision was made.

After exposing the skull, a craniotomy (~0.5×0.5mm) was made at approximately 5 mm posterior and 3.3 mm lateral from bregma to expose the transverse sinus. A Hamilton syringe (33-gauge needle) was inserted 3.3 mm lateral from the midline, 0.4 mm anterior to the transverse sinus and 2 mm deep from the brain surface. The Hamilton syringe was controlled by a Micro4 pump (World precision instruments), and injected 150 nl of virus at 10 nl/min. All animals were injected in the right hemisphere only. After completion of the injection, we waited for one minutes before partially (0.5 mm) retracting the needle, then after another 1-minute period the needle was fully retracted. The wound was rinsed and skin was sutured.

All injected animals were monitored for weight and behavioral deficits every day for a minimum of three days post-injection while receiving general analgesics. The animals that lost weight were at least monitored until they started regaining weight. All animals were first injected with the AAV 2.1-tetO-TVA-HA-csvG, and after 14 – 21 days injected with the recombinant rabies, and then perfused 5 days after the rabies injection.

### Immunohistochemistry

#### Tissue fixation and slicing

For anterograde viral tracing, after slice electrophysiology experiments, brain slices were drop-fixed in 4% paraformaldehyde (prepared from 16% aqueous solution; Electron Microscopy Sciences) in PBS and left overnight at 4°C. The slices were washed once with 0.3 M PBS-Glycine (Sigma Aldrich) for 15–20 minutes and three times in PBS. Slices were left in PBS at 4°C until immunostaining.

Animals that underwent freely moving behavior tests were deeply anesthetized with 5% isoflurane for 5 minutes, followed by an injection of 150 mg/kg and 10 mg/kg ketamine/xylazine. After checking for absence of reflexes, animals were transcardially perfused by inserting a 27-gauge needle into the left ventricle of the heart while simultaneously severing the right atrium. Following perfusion with 1X PBS, 4% paraformaldehyde in PBS was circulated in the animal. The brain was removed and fixed overnight in 4% paraformaldehyde at 4°C and then sectioned into 100 μm thick horizontal slices using a microtome (Leica VT1000S) after washing to undergo immunostaining.

For retrograde viral tracing, mice were perfused with and stored overnight in 4% paraformaldehyde at 4°C as described above. After fixation the brains were immersed in DMSO (2% dimethyl sulfoxide, VWR, cat. no. 23486.297; 20% glycerol, VWR, cat. no. 24387.292; 78% Phosphate Buffer 0.125 (PB)) for two nights to ensure cryoprotection before sectioning. The brains were mounted on a platform made with 30% sucrose (30 g sucrose, SIGMA, cat no. 16104–1KG; 100 ml PB), before being flash-frozen using dry ice. The tissue was sectioned into 4 series of 40 μm horizontal sections, using a Microm HM430 sliding microtome set to −32°C (Thermo Scientific, Waltham, MA). The first series from each brain was kept at 4°C pending immunohistochemistry, while the remaining tissue was immediately stored in DMSO at −20°C.

#### Immunostaining

Slices that underwent CUBIC tissue clearing^50^ were thoroughly washed with 0.1M PB and then moved to CUBIC#1 tissue clearing solution for two days. Next, slices were thoroughly washed in 0.1M PB before undergoing the standard immunostaining protocol. Slices were permeabilized in 0.5% PBS-Triton X (PBST), blocked in 3% Normal Goat Serum (NGS) for 2–4 hours, incubated with primary antibodies (overnight, 4°C), washed, and incubated with secondary antibodies (1–2 days, 4°C). The antibodies were diluted in blocking solution (1X PBS, 0.5% Triton, 3% NGS). After the final antibody incubation step, the slices were thoroughly washed in 0.1M PB and moved to CUBIC#2 tissue clearing solution for 1 hour, after which the slices were mounted on glass slides in CUBIC#2 solution. Slices that did not undergo tissue clearing immediately started with the standard immunostaining protocol, beginning with the permeabilization in 0.5% PBST and ending with the secondary antibody incubation step. These slices were then thoroughly washed in 1X PBS and mounted in Vectashield Hard Set Mounting Medium with DAPI (Vector Laboratories). For retrograde rabies virus tracing, immunostained slices were mounted in PB on positively charged slides and let dry for approximately 30 minutes. Upon drying, the slices were coverslipped with toluene and entellan and let dry overnight.

Primary antibodies used are: rabbit polyclonal anti-GFP primary antibody (1:1000, Invitrogen, lot 2185052), chicken anti-GFP (1:1000, Abcam, lot GR261775–1), guinea pig anti-MAP2 (1:1000, Synaptic Systems), rabbit polyclonal anti-WFS1 (1:2000, ProteinTech), mouse anti-NeuN (1:2000, Millipore, lot no 2819579), and anti-2A (1:1000, Merck Life Science AS (Millipore), lot no 2621952). Secondary antibodies used in anterograde experiments were raised in goat and procured from Invitrogen.

### Imaging

For all but retrograde rabies experiments, slices were imaged using an inverted Zeiss Axio Observer Z.1 Confocal Microscope, using (magnification/NA) 10x/0.3 air, 20x/0.8 air, and/or 40x/1.30 oil immersion objectives (Zeiss) and 405, 488, 594, and 647 nm lasers for excitation to acquire 1024 × 1024 pixel, 16-bit image stacks. Images were acquired every 5 μm (image stack step-size) for the 20x overview images to trace the hippocampal projections to different layers of entorhinal cortex and 1 μm for 40x images used for neuronal reconstructions. Tiled images were stitched using Zen Microscopy Software (Zeiss), and the tiled z-stacks were subsequently processed using Fiji (ImageJ).

For retrograde rabies virus tracing experiments, all processed tissue from each series was first scanned using a Zeiss Axio Scan.Z1 (ZEISS). Images were acquired with 20x objectives, single focus in the Z-plane. Slides were scanned using a quadruple filter (preset for DAPI, dl488, dl546 and dl647), using LEDs from a Calibri 2 source to excite each corresponding fluorophore.

### Slice preparation and electrophysiology

#### Slice preparation

Adult mice (12 – 14 week old) were deeply anesthetized with 5% isoflurane and transcardially perfused with ice-cold dissection artificial cerebrospinal fluid (dACSF) containing (in mM): sucrose 195.0, NaCl 10.0, glucose 10.0, NaHCO_3_ 25.0, KCl 2.5, NaH_2_PO_4_ 1.25, sodium pyruvate 2.0, CaCl_2_ 0.5, MgCl_2_ 7.0 with pH adjusted to 7.3, osmolarity of ~310 mOsm/l, and saturated with 95% O_2_ and 5% CO_2_. After decapitation, the brain was dissected out and quickly immersed in ice-cold dACSF. The olfactory bulbs and the cerebellum were separated from the brain block by two coronal cuts followed by a midsagittal cut to separate the two hemispheres. Using a brain block the two hemispheres were cut ventro-medially at a 10° angle. The two brain blocks were then glued (cyanoacrylate glue; Loctite 401, Henkel) to the stage of a VT 1200S vibratome (Leica microsystems, Wetzlar, Germany) such that the ventral side was glued on, the medial side was facing the blade and the dorsal side was facing upwards, submerged in ice-cold dACSF. For sectioning, the blade was positioned about 1 mm from the dorsal edge of the brain and sections were cut at a blade feed rate of 0.12 mm/s with an amplitude of 1.00 mm. Slices were incubated for 30 minutes in artificial cerebrospinal fluid (ACSF) containing (in mM): NaCl 125.0, glucose 22.5, NaHCO_3_ 25.0, KCl 2.5, NaH_2_PO_4_ 1.25, sodium pyruvate 3.0, ascorbic acid 1.0, CaCl_2_ 2.0, MgCl_2_ 1.0, with pH adjusted to 7.3, osmolarity of ~310 mOsm/l, and saturated with 95% O_2_ and 5% CO_2_. The ACSF was maintained at 35°C for the 30-minute incubation, and then kept at room temperature (22–24°C) until recording. All the chemicals were procured from Sigma Aldrich.

#### Electrophysiology

Patch-clamp recordings were made from pyramidal neurons (PNs) of EC_L2/3_ and EC_L5_ using a MultiClamp 700B amplifier (Axon Instruments) controlled by the pClamp 9 software (Molecular Devices), and junior micromanipulators on movable motorized shifting tables (Luigs & Neumann). Sampling interval and filter settings were 50 μs and 10 kHz respectively. Cells were visualized by differential interference contrast (DIC) microscopy through a 60× water-immersion objective (NA 1.0; Olympus) using an Olympus BX51 microscope and a Hamamatsu ORCA-R2 CCD camera using ImageJ Micromanager imaging acquisition software. All experiments were conducted at a temperature of 33–35°C, maintained by constant superfusion (flow rate 3–4 ml/min) of ACSF, heated by an inline solution heater (SH-27B with TC-324B controller; Warner Instruments, Hamden, CT, USA) and monitored by a thermistor placed between the inflow site and the slice in the recording chamber. In a subset of experiments, the ACSF was supplemented with 1 μM Tetrodotoxin citrate (TTX; HelloBio) and 100 μM 4-Aminopyridine (4-AP) to isolate monosynaptic connections.

Patch pipettes were pulled with P-1000 micropipette puller (Sutter Instruments Co., Novato, CA, USA) from borosilicate glass capillaries with filament (1.5 mm O.D. × 10 cm length × .86 mm I.D., Sutter Instruments BF150–86-10). Open tip pipette resistance was 3–4 MΩ when filled with intracellular solution containing (in mM): KMeSO_4_ 135 (for current clamp recordings) or CsMeSO_4_ 135 (for voltage clamp recordings), KCl 5, EGTA 0.1, HEPES 10, NaCl 2, Mg-ATP 5, Na_2_-GTP 0.4, Na_2_Phosphocreatine 10, and biocytin (4 mg/mL; 0.2%; Invitrogen) with a pH of 7.35 and an osmolarity of 300 mOsm/l. Current clamp recordings were obtained with access resistances up to 20 MΩ, compensated in bridge balance mode. The cells were maintained at a resting membrane potential of −70 mV by current injection to allow for the comparison of amplitudes of post-synaptic potentials (PSPs). For voltage – clamp recordings cells were held at either − 80 mV (to measure excitatory postsynaptic current; EPSC) or at + 10 mV (to measure inhibitory postsynaptic current; IPSC). Patch-clamped neurons with series resistance > 15 MΩ were discarded. All the chemicals were procured from Sigma Aldrich unless stated otherwise.

ChR2^+^ and JAWS^+^ hippocampal projections were optically stimulated Thorlabs LED system (470 and 625 nm wavelengths respectively). The axons of sensory input were minimally stimulated with a monopolar electrode in a patch pipette filled with ACSF, placed in EC_L1_ at a distance of at least 100 μm from the cell being recorded. Stimulating currents of 10–20 μA were delivered through a constant current isolated stimulator (Digitimer Ltd.).

During electrophysiological recordings, we checked every recorded neuron for functional ChR2 infection. To confirm adequate expression and function of ChR2 in a neuron we photostimulated the slice with a 500 ms light (470 nm) pulse while recording from the neuron in whole-cell configuration. The presence of a large, excitatory sustained photocurrent confirmed the presence of functional ChR2. Using this strategy, we ensured functional expression of ChR2 in CA1 pyramidal neurons. If any EC neuron produced a large sustained photocurrent upon photo stimulation, we discarded all the data from the animal.

In our recordings, we specially targeted PN in EC_L2/3_ and EC_L5_. We confirmed the identity of the PNs by their characteristic firing and sag properties, and neuronal morphologies ^37^. To measure firing and sag properties of the neurons, we injected depolarizing and hyperpolarizing currents respectively into the patch-clamped neurons held at −70 mV. All recordings we made at a holding potential of −70 mV for a reliable comparison of amplitudes of light evoked postsynaptic potentials (PSP). To obtain neuronal morphologies, every recorded neuron was filled with biocytin (mixed in with the intracellular solution in the recording pipette). Post-recording, the slices were fixed and immunostained with Streptavidin conjugated with a fluorophore for visualization. After imaging, the neuronal morphologies were reconstructed using the neuronal reconstruction software Neurolucida (MBF Bioscience).

To measure the input-output transformation of light-evoked response, the stimulation strength was incrementally adjusted (0–100% LED light intensity), keeping pulse duration (2 ms) constant. To measure peak PSP amplitudes, we recorded light-evoked responses every 15 seconds to a 2 ms light pulse (470 nm) delivered at 100% maximum LED strength while recording from EC_L2/3_ neurons, and at the highest LED strength that did not elicit AP firing while recording from EC_L5_ neurons. To isolate monosynaptic connections between the hippocampus and the entorhinal cortex, we recorded light-evoked responses from the EC neurons while washing in 1 μM TTX and 100 μM 4-AP with the recording ACSF. TTX blocks Na^+^ channels preventing AP firing, while 4-AP blocks K^+^ channels preventing repolarization of neuronal membrane. Together they prevent the firing and propagation of AP, blocking polysynaptic connections and isolating monosynaptic ones. To measure light-evoked excitatory and inhibitory postsynaptic currents neurons were held at −80 mV (reversal potential of GABA receptor mediated current) and +10 mV (reversal potential of glutamate receptor mediated current) respectively in voltage clamp configuration.

Long-term plasticity experiments were conducted in current clamp configuration. Pre– and post– induction postsynaptic potentials were recorded every 15 seconds. We photostimulated ChR2^+^ hippocampal projections using a 2 ms light pulse (470 nm) delivered at 100% maximum LED intensity while recording from EC_L2/3_ neurons and at the highest LED strength that did not elicit AP firing while recording from EC_L5_ neurons. We electrically stimulated of the sensory input entering at EC_L1_ with a short pulse of 0.1 ms and set the stimulus strength to elicit a subthreshold postsynaptic response reliably with each pulse. To pair the stimulation of the two inputs we first electrically stimulated (0.1 ms pulse) the sensory evoked input followed by the photostimulation (5 ms light pulse) of the hippocampal input after 20 ms. This pairing was repeated 90 times at 1 Hz.

### Modeling

We simulated a rate-based circuit with two sensory stimuli (s (i) = 1 if sensory input i is on, s (i) = 0 if off) which projected to a read-out neuron in EC_L2/3_, ro=w’ * s, where w are the weights from the sensory inputs onto the read-out neuron. The activity in the hippocampus, h, was initialised to 1 and received input projection from the read-out neuron with a synaptic weight of 1. The weights were potentiated with the pairing of the HC-EC_L2/3_ feedback projection and the sensory stimuli, w = w + α * h * s, where α= 0.005 is the learning rate (hippocampal feedback does not undergo plasticity). We initialised the weights to w = 1, and simulated 100 pairings where sensory input 1 (S_1_) was on. The activity of the read-out neuron, ro, was taken as a proxy for a behavioural read-out.

### in vivo freely moving behaviour

#### Novel Object Recognition Test (NOR)

This task is based on the natural and spontaneous behavior of rodents to spend more time exploring novel objects compared to familiar ones. For the first two days of the task, mice are habituated into the arena (length 37 cm, width 29 cm, height 27 cm) for 10 minutes without any object or cue. Locomotion distance is measured to verify that the viral infection and cannula implantation did not interfere with the behavior. On the third day (24 hour after the last habituation), the test started with two identical objects located in the arena (7 cm from the wall). For the acquisition/encoding phase, the mice were allowed to explore the identical objects for 10 minutes. After encoding, mice were returned to their home cages for 30 minutes (intersession interval (IVI)) for the consolidation period. After the IVI period, one of the objects was replaced with a different novel object, and the recall session started. During the recall session, laser light of 625 nm was delivered in control and JAWS injected animals. Here, mice were allowed to explore the arena with the familiar and novel the objects for 10 minutes.

#### Novel Object Location Test (NOL)

Similar to NOR, this task is based on the natural tendency of rodents to explore novel locations compared to familiar ones. Here, we used the same arena as was used in NOR task. Thus, the animals were already habituated during the first two days preceding the NOR task. On the fourth day (48 hours after the last habituation), NOL test began. The test started with two identical objects located in the arena in an equidistant and constant location (7 cm from the wall). For acquisition/encoding phase, mice were allowed to explore the objects in the constant location for 10 minutes. After encoding, mice were returned to their home cages for 30 minutes (intersession interval (IVI)) for the consolidation period. Following the IVI period, one of the objects was moved to new and constant location in the arena and recall session started. During the recall session, a laser light of 625 nm was delivered in control and JAWS injected animals. Here, mice were allowed to explore the arena with the familiar and novel location of the objects for 10 minutes.

#### Barnes Maze Test

Barnes maze is a test widely used to assess spatial memory encoding and recall related to hippocampal function. The equipment is a circular platform (90 cm in diameter), elevated 70 cm from the floor, with 20 holes (6 cm in diameter) located equidistant along its perimeter. Under one of the holes there is an escape box, located equidistant from the external cues placed around the experiment room. Cues are stable and maintained through the entire trial (training/encoding and test/recall).

The experiment consisted of seven days of training and one day of test. The training consisted of four trials/phases per day with an inter-trial interval of 15 minutes. At the beginning of each trial, mice were placed in the escape box for one minute. After that minute, mice were placed inside a black cylinder at the center of the platform for 10 seconds. Subsequently, the cylinder was removed and the mice were free to explore the platform for three minutes or until they found the escape box. If the mice were unable to locate the escape box within the allotted three minutes, they were gently guided to the escape box for another one minute and the trial ended. On the test day, the mice were located only in the center of the platform inside the cylinder for 10 seconds, the cylinder was removed and the mice were allowed to explore. All trials were conducted in presence of white light and noise (70 dB).

#### Optogenetic silencing during recall sessions

Animals were group housed as littermates before and after viral injection and cannula implants. During surgeries, littermate mice were assigned as JAWS and control. After 8 days of viral incubation, behavioral trials started. On the test day of NOR, NOL and Barnes maze, one 200 μm core multimode optic coupler fiber 50/50 (TT200FL1A) was connected with the bilateral implanted ceramic ferrules (Thorlabs, CFLC230–10) using a phosphor bronze split mating sleeve (Thorlabs, ADAL4–5). Red light (625 nm, Opto Engine LLC Diode Red 635nm Laser) was delivered through the fiber in 20 ms pulses at 20 Hz at an intensity of 10 3 mW for 10 minutes using a waveform generator (Siglent, SDG1032X). Measurement of the light intensity at the end of the fiber were done by an optical power meter (Thorlabs, PM100D) before every test experiment. Light stimulation was done in control and JAWs mice and bronze sleeves were covered with a dark tape to ensure the reproducibility of any light-induced behavioral changes.

### Data analysis

#### For anterograde viral tracing data

We used Fiji (ImageJ) to quantify fluorescence intensity of eYFP expressing hippocampal projections to the entorhinal cortex. On maximal projections of stacks of confocal images acquired as described above, regions of interest (ROI) were hand-drawn over the EC_L2/3_ and EC_L5_ to encompass areas receiving eYFP^+^ hippocampal projections. Integrated fluorescence intensity within EC_L5_ and EC_L2/3_ ROIs was calculated for all the slices (~6) along the dorsovental axis of the mouse brain. Ratio of mean fluorescence intensity represents the ratio of the mean integrated fluorescence intensity within the ROI drawn over EC_L5_ to that of EC_L2/3_ averaged across the dorsoventral axis of the mouse brain. To calculate the profiles plots of normalized integrated intensity of the hippocampal projections in the EC against the distance of the EC layer from pia we hand-drew a straight line across the EC layers from the edge of the pia to border between the EC and the hippocampus. The analysis was performed on maximal projections of stack of confocal images, and profile plot of fluorescence intensity were averaged across the dorsoventral axis of the mouse brain to obtain a mean profile plot for each animal.

#### For retrograde rabies virus tracing

##### Cell Counting –

Cells were counted with Neurolucida (MBF Bioscience) using live images from a Zeiss Axio Imager M2 (Carl Zeiss AG, Oslo, Norway). In order to acquire the data we used ultraviolet light (385 nm) to visualize DAPI through a 430 nm filter (Filter set 18, ZEISS) and blue (475 nm) light to excite the GFP signal through a 488 nm filter (Filter set 9, ZEISS) in the infected cells. The DAPI signal was used to create outlines of each section, and the GFP was used in order to visualize the cells in order to place location markers within the outline.

##### Cell criteria and projection criteria –

Cells were marked within the outline using a designated symbol, while projections were graded on a scale from 1–3 depending on volume and fluorescence intensity of the fibers. A cell was considered a cell when the GFP tag had filled the entire cell, so they had a pronounced soma as well as at least one neurite. In cases where it was uncertain, the cell was marked using a separate marker, and checked for co-localization with NeuN during cell counting.

Projections were included when the GFP signal was a continuous line (graded 1), when several axons ran along the same path (grade 2) or when the signal was so strong it saturated the area (grade 3).

In order to be able to later use the cell counts, vector files containing the outline for each section as well as location of each cell or projection was exported for each section in order to overlay the location of the cells with the NeuN and DAPI stains for delineation and cell counting.

##### Delineation of brain regions –

Scanned pictures were imported into Adobe Illustrator (Adobe) along with the vector files from Neurolucida. First the DAPI image was used to fit the vector file to the scans. Next the GFP image was overlaid to confirm the exact location of the markers, and to make fine adjustments of the image scale. Finally, the cell markers were placed in a separate layer and hidden, before overlaying the NeuN image in order to delineate the different areas. Delineation of the entorhinal and its neighboring areas of the hippocampal region was done according to the definitions set forth by (Blankvoort et al., 2018; M. P. Witter, 2010)^12,51^. Other areas were delineated using a combination of Paxinos’ The Mouse Brain in stereotactic coordinates (4th ed., 2012) atlas and Allen Brain Atlas. In some areas, such as with some of the midbrain nuclei, neither NeuN nor DAPI was able to yield convincing separation, and cells found in these areas were placed into categories based on their approximate location, for example the cells which were counted in raphe nuclei.

###### Delineating MEC_L3_ –

MEC Layer 3 is bordered by the lamina dissecans (layer 4) rostrally, which means this border is identified by the absence of cells in layer 4. MEC Layer 2 and 3 is delineated by the larger appearance of the layer 2 nuclei using a NeuN stain, compared to layer 3.

###### The MEC – Parasubiculum border -

The main points distinguishing MEC from parasubiculum using NeuN is that the large nuclei in MEC layer 2 widen toward the parasubicular border, before stopping. The lamina dissecans also becomes wider, while MEC layer 3 narrows. Additionally, the columnar organization of layer 5 ceases.

###### The Parasubiculum – Presubiculum border -

The border between pre- and parasubiculum is characterized by parasubiculum having slightly larger cells. Additionally, these borders were assessed based on the neighboring sections.

###### CA1-CA2 border -

The border of CA1-CA2 is hallmarked by the pyramidal cell layer becoming a bit thicker and less organized when compared to CA1. Since the NeuN signal does not provide a clear distinction between the areas, the DAPI signal was used for this border, where the DAPI forms a clear line throughout CA1, which becomes a lot less prominent as the PCP4 labelling starts in CA2. In the tissue stained for the CA2 marker PCP4 we can see that this border appears prominent throughout the dorsoventral axis (data not shown).

#### For ex vivo electrophysiology data

All the electrophysiology data were analyzed and graphs were generated using IgorPro (Wavemetrics) using custom written scripts. For all light-evoked post-synaptic responses, peak amplitude was measured as the maximum (for postsynaptic potentials and inhibitory postsynaptic current) or minimum (for excitatory postsynaptic current) from the baseline. The baseline value was averaged over 100 ms prior to stimulation onset (indicated by the stimulation artifact on representative traces). For the ITDP experiments, the pre-induction PSP amplitude was determined by averaging the steady state responses for 10 data points (3 mins) prior to pairing induction. Post-induction we recorded from each cell for at least 30 minutes or until the cell started firing action potentials consistently (which in EC_L2/3_ neurons occurred within the 5–10 minutes). To maintain uniformity in computing the potentiation and spike probability we used the last 10 data points of the recording post-induction. LTP was calculated by normalizing each sub-threshold post-induction PSP amplitude to the mean of the pre-induction steady state PSP amplitude. Spike probability was calculated as the probability of eliciting an action potential among the last 10 data points of the post-induction recording.

#### For in vivo freely moving behavior

All behaviors were video recorded from the top using an infrared camera (Basler, acA640–100gm) coupled to pylon Camera software for video acquisition at 20 frames per second in a semi-illuminated room. Behavioral analysis was performed and tracked after video acquisition, using ANY-maze video tracking software program (ANY-maze Stoeling Co.) for Novel Object Recognition (NOR) and Novel Object Location (NOL) tests. For NOR and NOL, analysis was defined as the exploration of the objects and object location during acquisition and recall phases when mice explored with the head directed and sniffing at a maximum distance of 2 cm from the objects.

The indices were calculated as follows:

Novel Object Recognition Index

$$\text{NOR}\;\text{Index}\;=\frac{\;\text{Novel}\;\text{Object}\;\text{exploration}\;\text{time}\;}{\;\text{Novel}\;\text{Object}\;+\;\text{Familiar}\;\text{Object}\;\text{exploration}\;\text{time}}$$

Novel Object Discrimination Index

$$\text{NOR}\;\text{Discrimination}\;\text{index}\;=\frac{\left(\;\text{Novel}-\;\text{Familiar}\;\text{object}\;\text{exploration}\;\text{time}\right)}{\left(\;\text{Novel}\;+\;\text{Familiar}\;\text{Object}\;\text{Exploration}\;\text{Time}\right)}$$

Novel Object Location Index

$$\text{NOL}\;\text{Index}\;=\frac{\;(\text{Novel}\;\text{location}\;\text{exploration}\;\text{time})\;}{\left(\;\text{Novel}\;+\;\text{Familiar}\;\text{location}\;\text{exploration}\;\text{time}\right)}$$

Novel Location Discrimination Index

$$\text{NOR}\;\text{Discrimination}\;\text{index}=\frac{\;(\text{Novel}\;-\;\text{Familiar}\;\text{location}\;\text{exploration}\;\text{time})\;}{\left(\;\text{Novel}\;+\;\text{Familiar}\;\text{location}\;\text{exploration}\;\text{time}\right)}$$

For Barnes maze test phase, analysis was done manually for number of errors and latency tracking. The number of errors represents the number of times the mice explored holes including the escape hole without entering and latency represents the total time that the animal takes to reach the escape hole.

### Statistical analysis

Data were analyzed using appropriate parametric or non-parametric tests, as stated in the text and figure legends. Normality of the data distribution was determined by Kolmogrov-Smirnov test, and the variance was compared using F-test. All normally distributed data with equal variance were tested for statistical significance using unpaired two-tailed students T-test. Normally distributed data with unequal variance was tested using unpaired two-tailed students T-test with Welch’s correction. Non-normally distributed data were tested for statistical significance with Wilcoxon’s signed rank test. Data are shown as mean ± SEM with median unless otherwise stated. Significance level was set at p < 0.05. Prism (GraphPad) was used for plotting data and statistical analyses. Figures were generated using Adobe Illustrator.

## Extended Data

**Extended Data Fig. 1.**
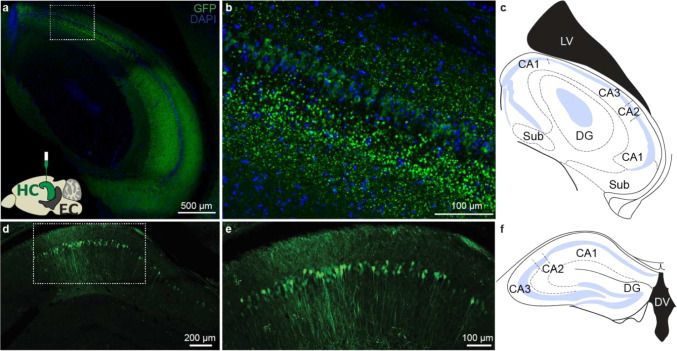
Injection site in dorsal hippocampus CA1. a. Confocal image of a horizontal brain slice of a mouse injected with AAV 2.5 EF1α-double floxed-ChR2-eYFP into dorsal CA1 and immunostained for GFP to demarcate the injection site and DAPI (nuclear marker). Bottom inset shows a sagittal profile of a mouse brain depicting the injection strategy. CamKII Cre or CamKII Cre x Ai14 mice were injected with AAV 2.5 EF1α-double floxed-ChR2-eYFP into dorsal CA1.The area within the white dotted box is expanded in panel b. b. Magnified view of the infected CA1 neurons within the white dotted box in panel a. c. Schematic adapted from Paxinos mouse brain atlas depicting a horizontal section of a mouse brain showing the regions within the hippocampal formation: areas CA1, CA2, CA3, dentate gyrus (DG), and subiculum (Sub), and lateral ventricle (LV). d. Confocal image of a coronal brain slice of a mouse injected with AAV 2.2 CAMKII-ChR2-eYFP into dorsal CA1 and immunostained for GFP to demarcate the injection site. The area within the white dotted box is expanded in panel e. e. Magnified view of the infected CA1 neurons within the white dotted box in panel d. f. Schematic adapted from Paxinos mouse brain atlas depicting a coronal section of a mouse brain showing the regions within the hippocampal formation: areas CA1, CA2, CA3, dentate gyrus (DG), and dorsal 3^rd^ ventricle (DV).

**Extended Data Fig. 2.**
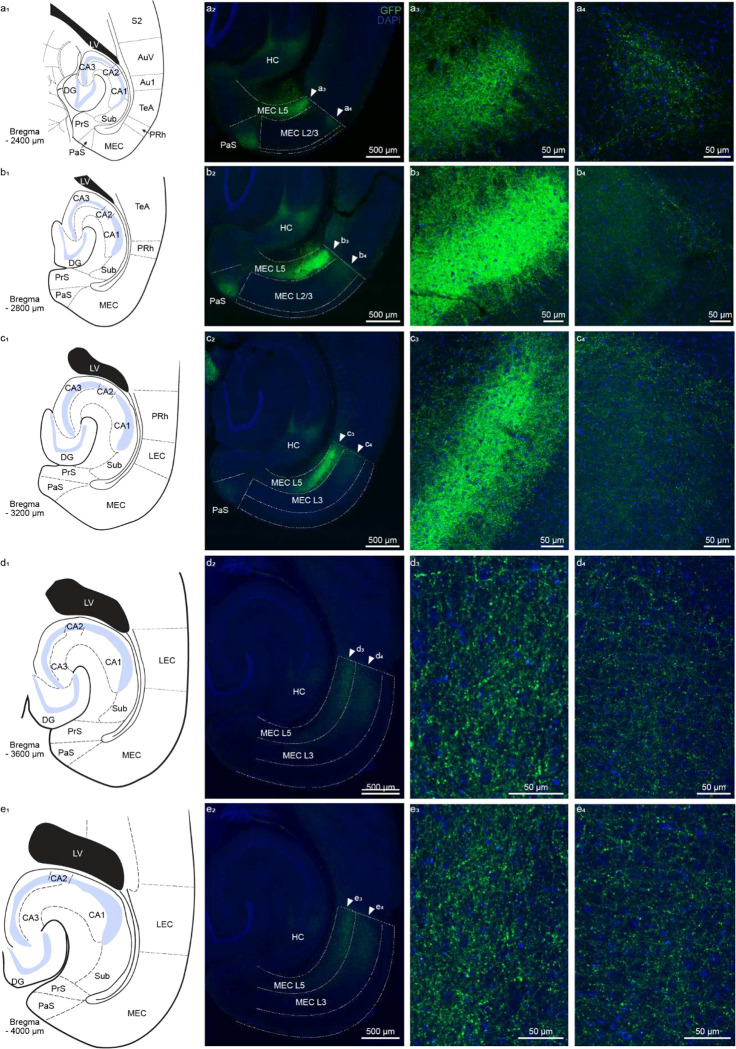
Dorsal to ventral axis of hippocampal projections to entorhinal cortex. a. (a_1_) Schematic adapted from Paxinos mouse brain atlas depicting a horizontal section of a mouse brain showing the various brain regions that are included in the confocal image in panel a_2_. The position of the horizontal plane of section through the mouse brain is reported as ventral distance from bregma (−2400 μm) in the bottom left corner. (a_2_) Confocal image of horizontal brain slice of a mouse injected with AAV2.5 EF1α-double floxed-ChR2-eYFP into dorsal CA1 and immunostained for GFP and DAPI (nuclear marker). Green hippocampal axons were observed in medial entorhinal cortex (MEC) layers 5 and 2/3, and parasubiculum. White dotted lines demarcate the different cortical layers and brain regions. Arrowheads point the layer detailed in panels a_3_ and a_4_. (a_3_) Magnified view of the hippocampal axons in EC_L5_, an area pointed by the white arrowhead labeled a_3_ in panel a_2_. (a_4_) Magnified view of the hippocampal axons in EC_L2/3_, an area pointed by the white arrowhead labeled a_3_ in panel a_2_. b. Same as a. but for −2800 μm from bregma. c. Same as a. but for −3200 μm from bregma. d. Same as a. but for −3600 μm from bregma. e. Same as a. but for −4000 μm from bregma. Abbreviations used: Au1 – primary auditory cortex, AuV – secondary auditory cortex ventral, DG – dentate gyrus, LEC – lateral entorhinal cortex, LV – lateral ventricle, MEC – medial entorhinal cortex, PaS – parasubiculum, PRh – perirhinal cortex, PrS – presubiculum, S2 – secondary somatosensory cortex, Sub – subiculum, TeA – temporal association cortex.

**Extended Data Fig. 3.**
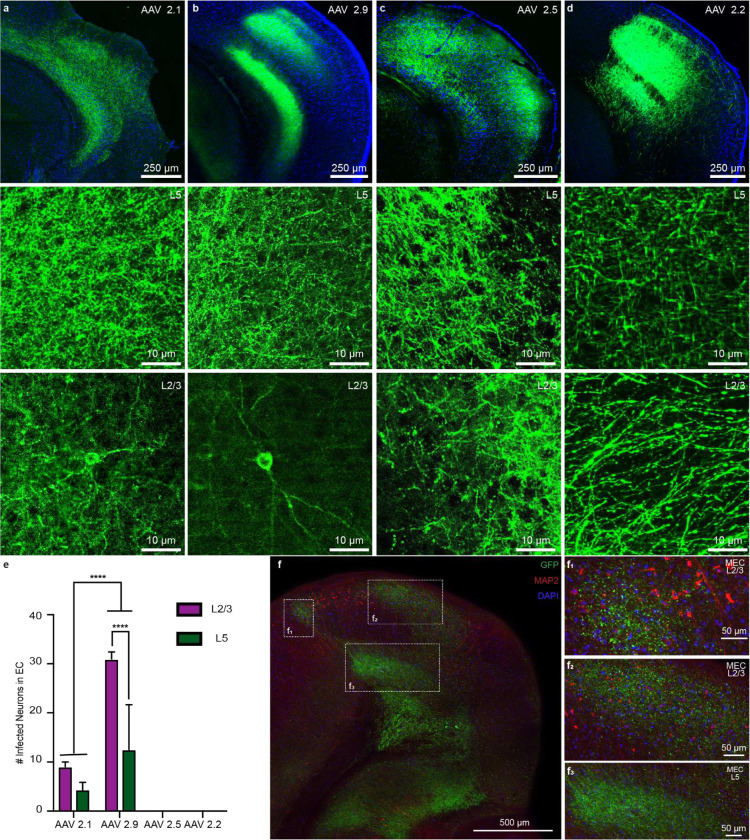
Validation of hippocampal feedback using different viral strategies. a. *Top -* Confocal image of a horizontal brain slice of a mouse injected with AAV2.1 EF1α-double floxed-ChR2-eYFP (41.5 nl/site with 3 week incubation) into dorsal CA1 and immunostained for GFP and DAPI (nuclear marker). Green hippocampal axons were observed in entorhinal cortex layers 5 and 2/3. *Center –* Expanded view of the hippocampal projection to EC_L5_. No infected neurons were observed in EC_L5_. *Bottom –* Expanded view the hippocampal projection to EC_L2/3_ showing a sample infected EC_L2/3_ neuron. b. *Top –* Confocal image of a horizontal brain slice of a mouse injected with AAV2.9 EF1α-double floxed-ChR2-eYFP (41.5 nl/site with 3 week incubation) into dorsal CA1 and immunostained for GFP and DAPI (nuclear marker). Green hippocampal axons were observed in entorhinal cortex layers 5 and 2/3. *Center –* Expanded view of the hippocampal projection to EC_L5_. No infected neurons were observed in EC_L5_. *Bottom –* Expanded view the hippocampal projection to EC_L2/3_ showing a sample infected EC_L2/3_ neuron. c. *Top –* Confocal image of a horizontal brain slice of a mouse injected with AAV2.5 EF1α-double floxed-ChR2-eYFP (82.8 nl/site with 3 week incubation) into dorsal CA1 and immunostained for GFP and DAPI (nuclear marker). Green hippocampal axons were observed in entorhinal cortex layers 5 and 2/3. *Center –* Expanded view of the hippocampal projection to EC_L5_. *Bottom –* Expanded view the hippocampal projection to EC_L2/3_. No infected neurons were observed in either EC layer. d. *Top –* Confocal image of a horizontal brain slice of a mouse injected with AAV2.2 CAMKII-ChR2-eYFP (41.4 nl/site with 3 week incubation) into dorsal CA1 and immunostained for GFP and DAPI (nuclear marker). Green hippocampal axons were observed in entorhinal cortex layers 5 and 2/3. *Center –* Expanded view of the hippocampal projection to EC_L5_. *Bottom –* Expanded view the hippocampal projection to EC_L2/3_. No infected neurons were observed in either EC layer. e. Quantification of the number of infected neurons observed in EC after 3 weeks of viral incubation period. 41.4 nl of virus was injected per site for viral serotypes AAV 2.1 (n = 8), 2.2 (n = 8), and 2.9 (n = 22), and 82.8 nl per site for AAV 2.5 (n = 11). f. Confocal image of a horizontal brain slice of a mouse injected with AAV 2.5 EF1α-double floxed-ChR2-eYFP into dorsal CA1 and immunostained for GFP, MAP2 (marker for dendrites), and DAPI (nuclear marker). Green hippocampal axons were observed in entorhinal cortex layers 5 and 2/3. The area within the white dotted boxes are expanded in panels f_1_, f_2_, and f_3_. Absence of colocalization between the GFP and MAP2 immunofluorescence signals verify the absence viral infection in EC neurons.

**Extended Data Fig. 4.**
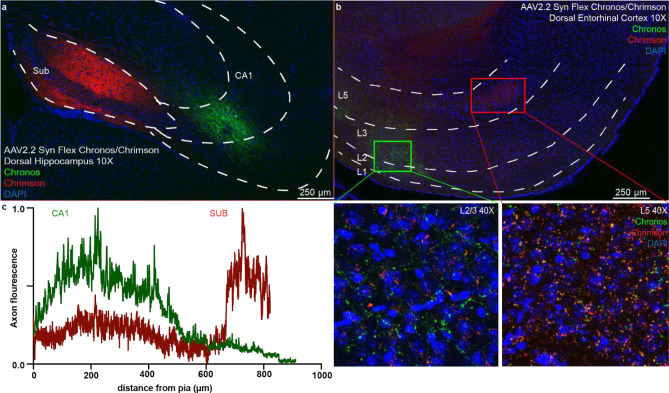
Hippocampal CA1 primarily projects to entorhinal cortex layer 2/3 while subiculum projects to layer 5. a. Confocal image of a horizontal brain slice of a mouse injected with AAV 2.2 Syn flex Chrimson TdTom in dorsal subiculum and AAV 2.2 Syn flex Chronos GFP in dorsal CA1, immunostained for GFP, RFP, and DAPI. White dotted lines demarcate the different hippocampal areas: Sub – Subiculum, and CA1- CA1 pyramidal layer. b. Confocal image of a horizontal brain slice of a mouse injected with AAV 2.2 Syn flex Chrimson TdTom in dorsal subiculum and AAV 2.2 Syn flex Chronos GFP in dorsal CA1, immunostained for GFP, RFP, and DAPI. Green CA1 axons were primarily observed in entorhinal cortex (EC) layers 2/3, while red subiculum axons in EC layer 5. White dotted lines demarcate the different cortical layers. Projections to EC_L2/3_ and EC_L5_ enclosed in green and red boxes respectively are magnified in bottom left and bottom right insets respectively. c. Normalized fluorescence intensity of hippocampal axons in EC as a function of distance from pia. Red and green traces represent the fluorescence along the axons from subiculum and CA1 respectively. In total 6 animals were used to resolve HC-EC_L2/3_ and HC-EC_L5_ pathways.

**Extended Data Fig. 5.**
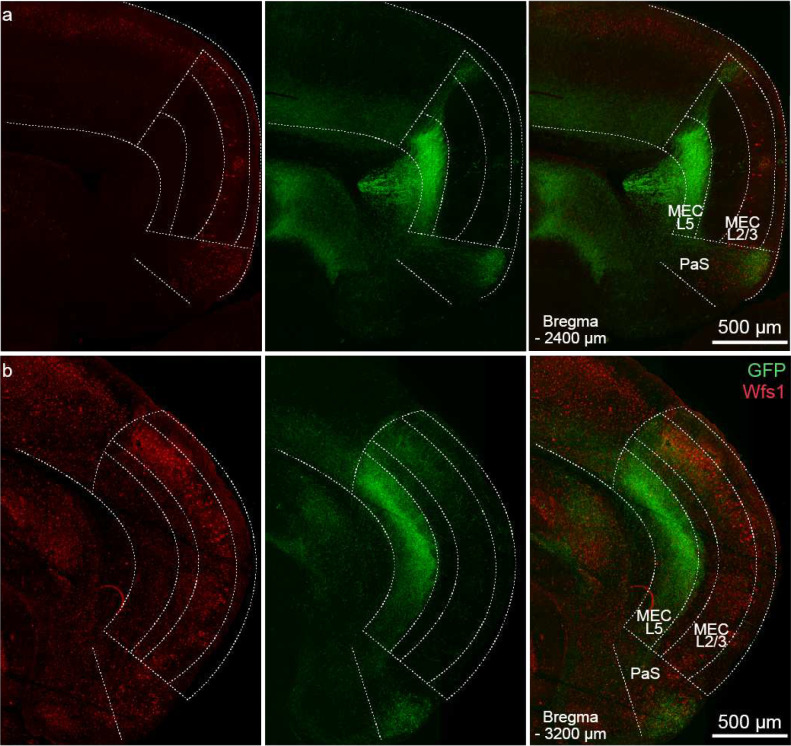
Hippocampal projections primarily target medial entorhinal cortex (MEC). a. Confocal image of horizontal brain slice of a mouse injected with AAV2.5 EF1α-double floxed-ChR2-eYFP into dorsal CA1 and immunostained for GFP and Wfs1 (labels pyramidal cells in MEC). White dotted lines demarcate the different cortical layers and brain regions. Note the sharp cut-off of green hippocampal projections at the MEC border demarcated by the absence of clusters of pyramidal cells labeled by Wfs1. The position of the horizontal plane of section through the mouse brain is reported as ventral distance from bregma (−2400 μm) in the bottom left corner. PaS – parasubiculum. b. Same as a. but for −3200 μm from bregma.

**Extended Data Fig. 6.**
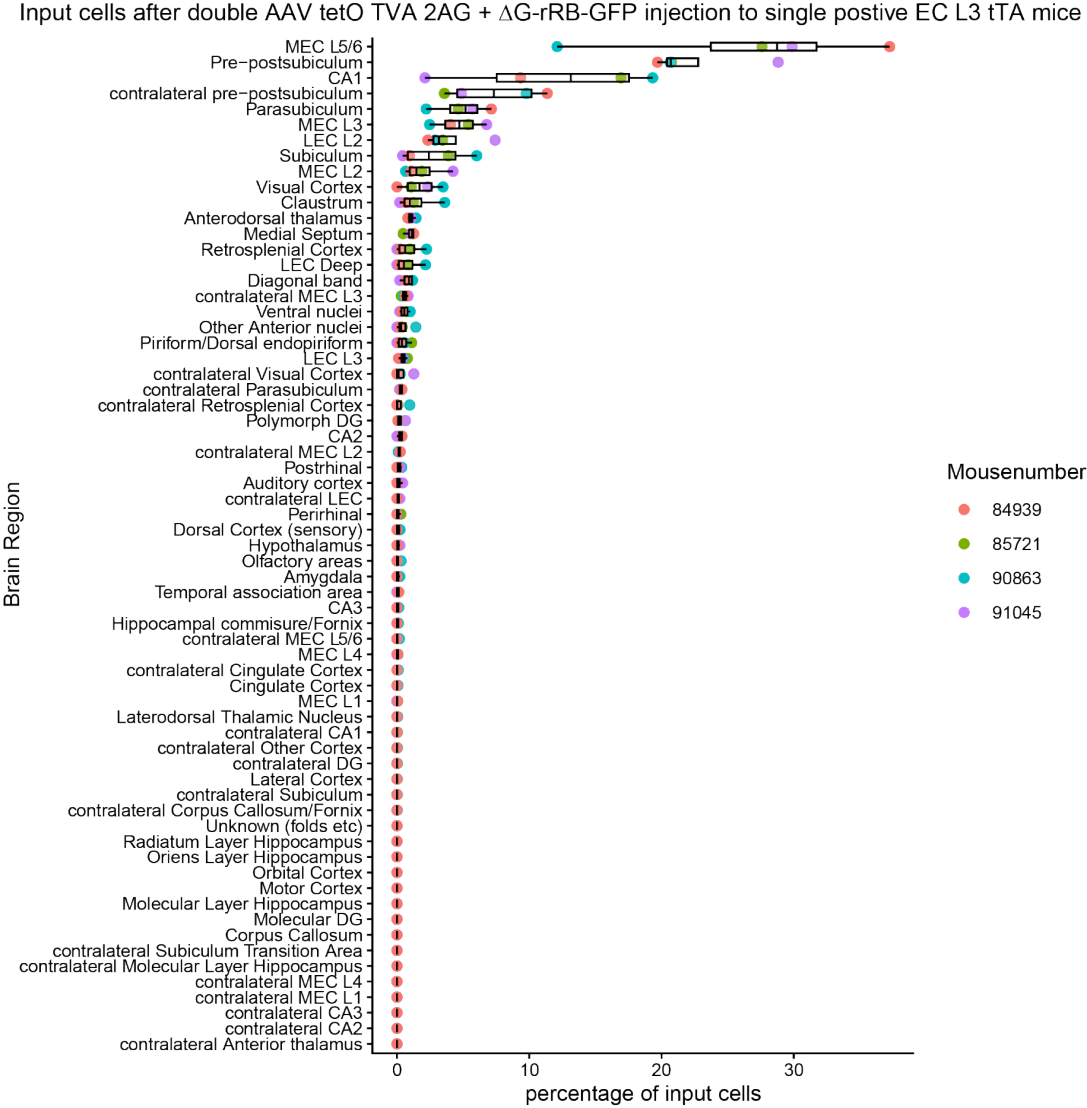
Quantification of GFP^+^ presynaptic cells. Quantification of GFP^+^ presynaptic input cells in brain regions that project to starter neurons in MEC_L2/3_ shown by double injection of AAV tetO TVA 2AG and ΔG-rRB-GFP to EC L3 tTA mice. Abbreviations used: DG – dentate gyrus, LEC – lateral entorhinal cortex, MEC – medial entorhinal cortex.

**Extended Data Fig. 7.**
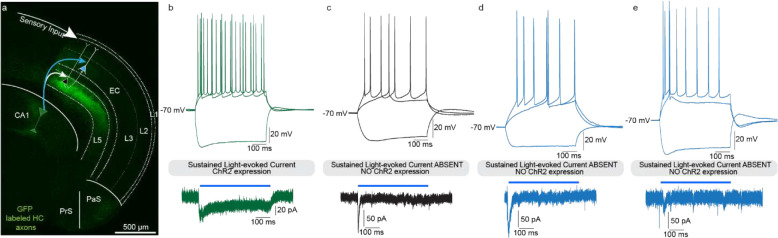
Functional channelrhodopsin 2 (ChR2) expression only in hippocampal neurons and axonal projections. a. Schematic of the proposed circuit overlaid on a confocal image of a horizontal mouse brain slice. Green hippocampal projections from hippocampal CA1 project not only to EC_L5_ but also to EC_L2/3_. EC – entorhinal cortex, PrS – presubiculum, PaS – parasubiculum. b. *Top* – Fire and sag properties recorded from a CA1 pyramidal neuron. Shown here are the intrinsic electrical responses of the neuron to current injection of −240 pA, and current corresponding to rheobase, and 1.5 x rheobase. *Bottom* – Sustained photocurrent elicited in response to a 500 ms 470 nm light pulse demonstrates the expression of functional ChR2 in the CA1 pyramidal neuron. c. *Top* – Fire and sag properties recorded from an EC_L2/3_ stellate cell. Shown here are the intrinsic electrical responses of the neuron to current injection of −240 pA, and current corresponding to rheobase, and 1.5 x rheobase. *Bottom* – Absence of sustained photocurrent elicited in response to a 500 ms 470 nm light pulse confirms the lack of off-target viral infection in the EC leading to ChR2 expression. d – e. Same as c but for ECL_2/3_ (d) and EC_L5_ (e) pyramidal neurons.

**Extended Data Fig. 8.**
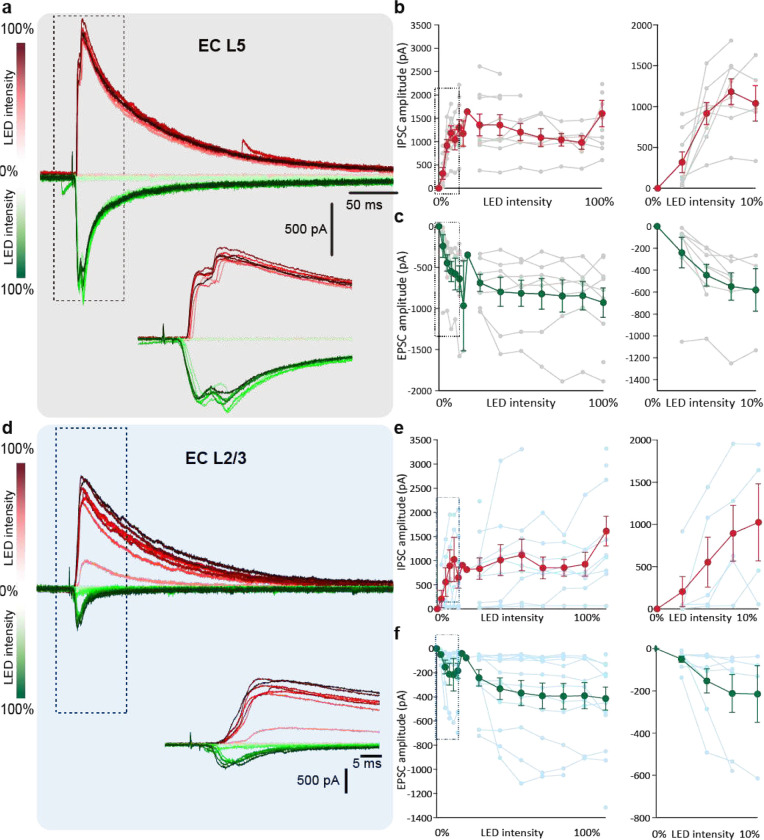
Input – output transformation of light – evoked postsynaptic currents at EC neurons upon increasing strength of optical stimulation of ChR2^+^ hippocampal axons. a. Representative voltage clamp recordings from EC_L5_ neuron to record excitatory and inhibitory postsynaptic currents (EPSC and IPSC respectively) at −80 mV and +10 mV respectively. Insets show parts of the trace enclosed in the dotted box. b. IPSC amplitudes recorded at EC_L5_ neurons in response to increasing LED intensity for the 470 nm optical stimulation of ChR2^+^ hippocampal projections to EC. Each gray trace is from a single neuron and the bold red trace represents the mean ± SEM across neurons. Data points within the black dotted box in the *left* panel are expanded in the *right* panel. c. Same as b. but for EPSC amplitudes recorded at EC_L5_ neurons. d. Same a. but for EC_L2/3_ neurons. e. Same as b. but for EC_L2/3_ neurons. f. Same as b. but for EPSC amplitudes recorded at EC_L2/3_ neurons.

**Extended Data Fig. 9.**
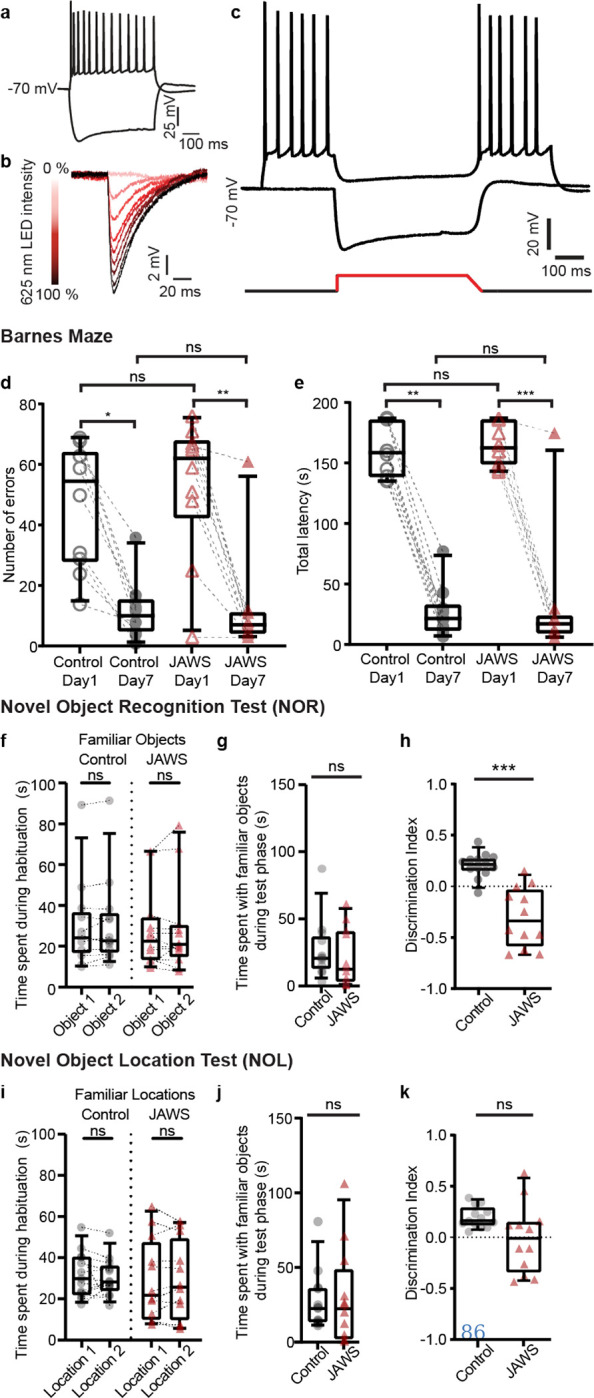
Validation *in vivo* optogenetic silencing of the HC-EC_L2/3_ circuit. a – c. Validation of JAWs silencing in acute mouse brain hippocampal slices. (a) Characteristic firing and sag pattern of a CA1 pyramidal neuron (PN). (b) Step-increase in hyperpolarization in response to pulses of increasing intensity of 625 nm LED. (c) *Top – v*alidation of far-red light activated inhibitory opsin JAWs silencing. CA1 PN stays at −70 mV in the absence of current injection and fires characteristically upon depolarizing current (300 pA) current injection, except when it hyperpolarizes in the 500 ms interval of JAWs activating 625 nm light stimulation (*top*). *Bottom – s*timulation pattern: 500 ms of 625 nm light stimulation in the middle of a second-long recording period (*bottom*). d. Comparison of the total number of errors made by the animal during the learning phase on day 1 vs day 7. Errors were counted as the number of times an animal explored different holes including the escape hole without entering the escape box. Box and whisker plots show the median number of errors made by the cohort of animals (control – black, JAWS – red), lower/upper quartile, 10–90^th^ percentiles. Each gray dot and red triangle represents the number of errors made by an individual control and JAWs animal respectively. Statistical significance tested with Kruskal-Wallis test with post-doc Dunn’s correction multiple comparisons. p – value * = 0.02, ** = 0.002, ns < 0.05. e. Comparison of the total time for which the animal explored the arena (latency) before entering the escape box, during the learning phase on day 1 vs day 7. Box and whisker plots show the median latency of entering the escape box by the cohort of animals (control – black, JAWS – red), lower/upper quartile, 10–90^th^ percentiles. Each gray dot and red triangle represents the latency of an individual control and JAWs animal respectively. Statistical significance tested with Kruskal-Wallis test with post-doc Dunn’s correction multiple comparisons. p – value ** = 0.005, *** = 0.0004, ns < 0.05. f. Comparison of the time that the animals spent exploring the two familiar and similar objects during habituation phase of NOR task. Box and whisker plots show the median exploration time of the cohort of animals during familiar trial (control – black, JAWS – red), lower/upper quartile, 10–90^th^ percentiles. Each gray dot and red triangle represents the exploration time of an individual control and JAWs animal respectively. Statistical significance tested with Wilcoxon matched-pairs signed rank test for both cohorts, p – value = 0.3308 for control, p – value > 0.9999 for JAWs injected animals. g. Comparison of the time that the animals spent exploring the familiar objects during NOR test trial. Box and whisker plots show the median exploration time of the cohort of animals during the NOR test trial (control – black, JAWS – red), lower/upper quartile, 10–90^th^ percentiles. Each gray dot and red triangle represents the exploration time of an individual control and JAWs animal respectively. Statistical significance tested with Mann Whitney test, p – value = 0.2471. h. Box and whisker plots showing the median novel object discrimination index for the cohort of animals (control – black, JAWS – red), lower/upper quartile, 10–90^th^ percentiles during NOR test trial. Each gray dot and red triangle represents the novel object discrimination index an individual control and JAWs animal respectively. Statistical significance tested with unpaired t test with Welch’s correction, p – value < 0.0001. i. Comparison of the time that the animals spent exploring the two familiar and similar objects during habituation phase of NOL task. Box and whisker plots show the median exploration time of the cohort of animals during familiar trial (control – black, JAWS – red), lower/upper quartile, 10–90^th^ percentiles. Each gray dot and red triangle represents the exploration time of an individual control and JAWs animal respectively. Statistical significance tested with paired t-test for both cohorts, p – value = 0.5131 for control, p – value = 0.9661 for JAWs injected animals. j. Comparison of the time that the animals spent exploring the familiar locations during NOL test trial. Box and whisker plots show the median exploration time of the cohort of animals during the NOL test trial (control – black, JAWS – red), lower/upper quartile, 10–90^th^ percentiles. Each gray dot and red triangle represents the exploration time of an individual control and JAWs animal respectively. Statistical significance tested with Mann Whitney test, p – value = 0.7689. k. Box and whisker plots showing the median novel location discrimination index for the cohort of animals (control – black, JAWS – red), lower/upper quartile, 10–90^th^ percentiles during NOR test trial. Each gray dot and red triangle represents the novel location discrimination index an individual control and JAWs animal respectively. Statistical significance tested with unpaired t test with Welch’s correction, p – value = 0.0626.

## Figures and Tables

**Fig. 1.**
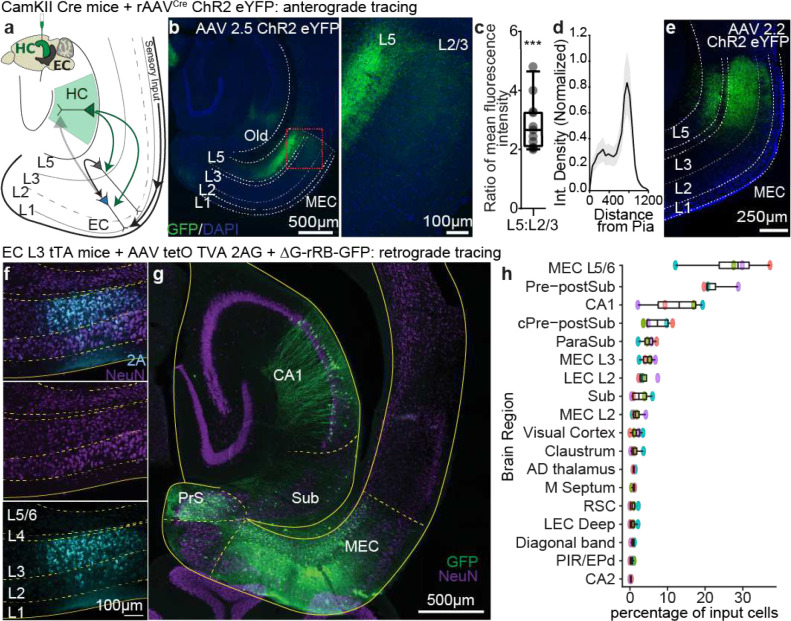
Anterograde and retrograde viral tracing identifies a novel hippocampal feedback to superficial layers of entorhinal cortex. a. Schematic showing the injection strategy (top inset) and the proposed novel circuit. Injection strategy – Sagittal profile of a mouse brain showing hippocampus (HC) in green and entorhinal cortex (EC) in black. CamKII Cre or CamKII Cre x Ai14 mice were injected with AAV 2.5 EF1α-double floxed-ChR2-eYFP into dorsal CA1. Proposed circuit model - a horizontal slice through the mouse brain shows eYFP expressing hippocampal axons (green arrows) project to not only the neurons in EC_L5_ but also to EC_L2/3_, the layer which sends direct cortical feed to the HC (gray arrow). EC_L5_ projects to EC_L2/3_ (black arrow) while the sensory input from primary sensory cortices enters at EC_L1_. b. *left -* Confocal image of hippocampal projections to medial entorhinal cortex (MEC) in a 400 μm horizontal section through the brain of a CamKII Cre mouse injected with AAV 2.5 EF1α-double floxed-ChR2-eYFP into dorsal CA1. The brain slice was stained for GFP to demarcate the hippocampal feedback projections to deep EC_L5_ and to superficial EC_L2/3_, and DAPI (nuclear marker). White dotted lines demarcate the different cortical layers. The area within the red dotted box is expanded on the *right* – Magnified view of the hippocampal projections to EC_L5_ and ECL_2/3_ within the red dotted box in the left panel. The hippocampal projections at EC_L2/3_ appear to have lower immunofluorescence intensity as compared to the projections at EC_L5_. c. Ratio of mean fluorescence intensity of axonal projections to EC_L5_ to EC_L2/3_ presented as a box and whisker plot (median intensity, lower/upper quartile, 10–90^th^ percentiles). Each data point represents one animal. Statistical significance tested with one-sample Wilcoxon signed rank test, p = 0.0010, n = 11. d. Normalized fluorescence intensity of hippocampal axons in EC as a function of distance from pia (n = 11). Bold line flanked by shaded area denote mean fluorescence intensity and SEM respectively. e. Confocal image of hippocampal projections to medial entorhinal cortex (MEC) in a 400 μm horizontal section through the brain of a CamKII Cre mouse injected with AAV 2.2 CAMKII-ChR2-eYFP into dorsal CA1. The brain slice was stained for GFP to demarcate the hippocampal feedback projections to deep EC_L5_ and to superficial EC_L2/3_, and DAPI (nuclear marker). White dotted lines demarcate the different cortical layers. The mean immunoflurorescence intensity of hippocampal projection in EC_L2/3_ (0.43 ± 0.02 a.u.) was half the immunoflurorescence intensity of hippocampal projection in EC_L5_ (0.83 ± 0.01 a.u.), two-tailed students t-test, p < 0.0001, n = 8. f. Local expression of “TVA-2A-rabies G protein” after injection of AAV-tetO-TVA 2AG to EC_L2/3_. 2A positive cells were found primarily in EC_L3_ and in smaller amounts in EC_L2_, but rarely outside of these two layers. g. GFP expression in presynaptic cells after injection with AAV-tetO-TVAG followed by ΔG-rRB-GFP. Presynaptic cells were primarily found in deep layers of MEC, Presubiculum (PrS), and CA1. h. Quantification of GFP^+^ (presynaptic) cells in brain regions that project to starter neurons in MEC_L2/3_. MEC L5/6 – medial entorhinal cortex layer 5/6, pre-postSub – pre-post Subiculum, cPre-postSub – contralateral pre-post subiculum, paraSub – parasubiculum, MEC L3 – MEC layer 3, LEC L2 – lateral entorhinal cortex layer 2, sub – subiculum, MEC L2 – MEC layer 2, AD thalamus – anterodorsal thalamus, M septum – medial septum, RSC – retrospenial cortex, PIR/EPd – piriform cortex/ dorsal endopiriform.

**Fig. 2.**
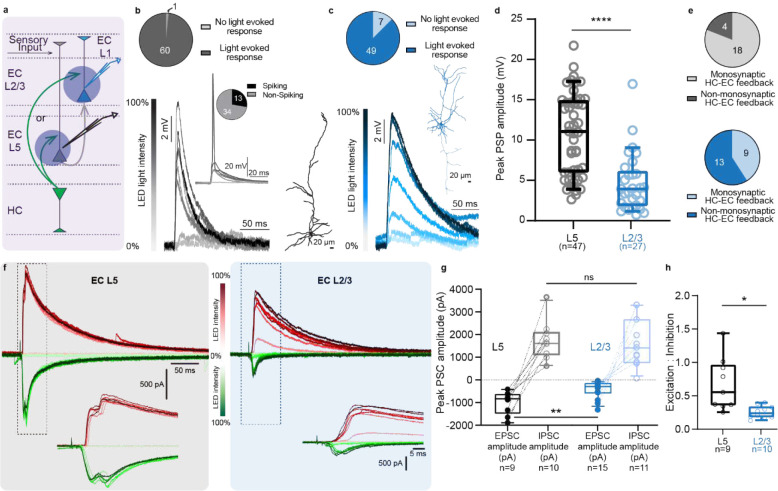
Hippocampal feedback recruit strong excitation upon EC_L5_ neurons, but predominantly feed-forward inhibition upon EC_L2/3_ neurons. a. Schema of physiology experiments to parse the HC-to-EC reciprocal feedback. ChR2 expressing hippocampal axons (green arrows) were optically stimulated with 470 nm light pulse (2 ms; blue circles) while light-evoked electrical responses are recorded from patch-clamped neurons in EC_L5_ or EC_L2/3_. b – c. HC-EC_L5_ feedback has a higher probability of a functional connection and greater synaptic strength than HC-EC_L2/3_. Light-evoked response was detected in 60/61 (98.36 %) EC_L5_ neurons when optically stimulating ChR2^+^ HC projections in EC_L5_ (b *top*), with 13/47 (27.66%) of neurons firing action potentials (AP; b *inset*). Whereas, optical stimulation of hippocampal projections elicited light evoked responses in 49/56 (87.50 %) EC_L2/3_ neurons (c *top*), but never led to AP firing. Sample traces of post-synaptic potentials (PSPs) in EC_L5_ (black; b *bottom*) and EC_L2/3_ (blue; c *bottom*) neurons with increasing LED light intensity. Each recorded cell was filled with biocytin and immunostained post-hoc using streptavidin conjugated with a fluorophore for visualization. Reconstructed neuronal morphology, and firing and sag properties were used to identify pyramidal neurons in EC_L5_ (black cell fill in b) and EC_L2/3_ (blue cell fill in c). d. Peak amplitudes of light-evoked PSPs in EC_L5_ (black) vs. EC_L2/3_ (blue) neurons presented as box and whisker plots (median intensity, lower/upper quartile, 10–90^th^ percentiles). Each data point represents mean peak PSP amplitude of an individual neuron. Statistical significance tested with Mann Whitney test, p<0.0001. Data from only pyramidal neurons both in EC_L5_ and EC_L2/3_ were analyzed. e. Higher probability of monosynaptic connection between the HC and EC_L5_ than HC and EC_L2/3_ determined by the persistence of light-evoked responses even in the presence of TTX and 4-AP that block AP and membrane repolarization respectively, hence blocking polysynaptic response. 18/22 (81.82 %) EC_L5_ neurons (black) were monosynaptically connected to HC (*top*) while only 9/22 (40.91 %) EC_L2/3_ (blue) received direct monosynaptic HC feedback (*bottom*). f. Representative traces of excitatory and inhibitory postsynaptic currents (EPSC and IPSC respectively) recorded at −80 mV and +10 mV respectively from voltage clamped EC_L5_ (*left*) and EC_L2/3_ (*right*) neurons. Insets show parts of the traces enclosed in the dotted box. g. EC_L5_ and EC_L2/3_ neurons have comparable light-evoked IPSC amplitudes but EPSC amplitude at HC-EC_L5_ synapse is significantly greater than at HC-EC_L2/3_ synapse. Peak amplitudes of PSCs in EC_L5_ (EPSC in black; IPSC in gray) vs. EC_L2/3_ (EPSC in blue; IPSC in light blue) neurons presented as box and whisker plots (median intensity, lower/upper quartile, 10–90^th^ percentiles). Each data point represents mean peak PSC amplitude of an individual neuron. Statistical significance tested with unpaired t-test, ns- not significant, **p = 0.005. Data points connected by dotted lines represent a subset of neurons from which both EPSCs and IPSCs were recorded. h. Excitation to inhibition (E/I) ratio is significantly smaller at HC-EC_L2/3_ than at HC-EC_L5_. E/I ratio in EC_L5_ (black) vs. EC_L2/3_ (blue) neurons presented as box and whisker plots (median intensity, lower/upper quartile, 10–90^th^ percentiles). Each data point represents the E/I ratio of an individual neuron. Statistical significance tested with unpaired t-test with Welch’s correction, *p = 0.01. Only the neurons from which both EPSC and IPSC were recorded (represented as data points connected by dotted lines in panel g) were included in the E/I ratio analysis.

**Fig. 3.**
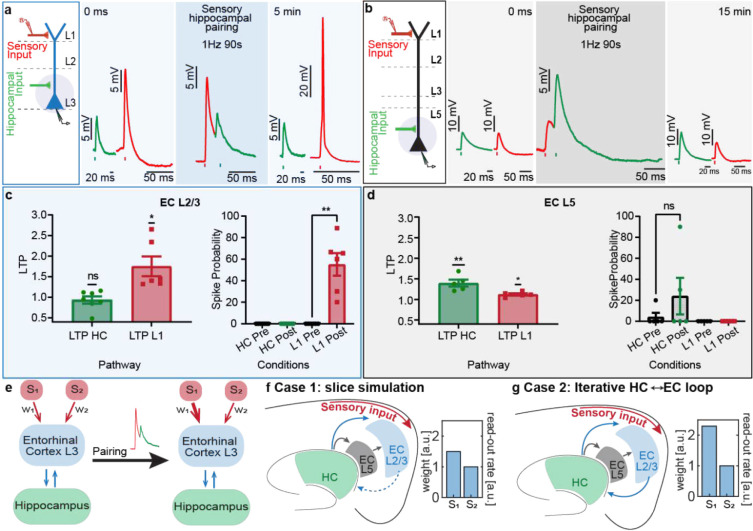
HC-EC_L2/3_ feedback heterosynaptically modulates the sensory output in the EC. a – b. Schematic showing experimental protocol – optical activation of hippocampal inputs (green) was coupled to the electrical stimulation of sensory inputs (red) at an interval of 20 ms for 90 s at 1 Hz in EC_L2/3_ (a) and EC_L5_ (b) neurons. c. Post coupling, the sensory evoked response in EC_L2/3_ significantly potentiates (one sample Wilcoxon signed rank test, p – value = 0.03) while the hippocampal input evoked response remains unchanged (one sample t-test, p – value = 0.48). Sensory evoked response in EC_L2/3_ also shows an increased probability to generate action potentials (AP; Wilcoxon matched-pairs signed rank test, p – value = 0.03) with no change in the hippocampal input evoked spike probability (both pre- and post-coupling spike probability = 0). d. Post coupling, both the hippocampal (one sample t-test, p – value = 0.0094) and the sensory input evoked responses (one sample t-test, p – value = 0.011) show significant potentiation at EC_L5_ neurons but there is no significant change in AP firing probability in either case (HC evoked response: Wilcoxon matched-pairs signed rank test, p – value = 0.50; sensory input evoked response: both pre- and post-coupling spike probability = 0). e. Schema of the rate based circuit model comparing the weights (w_1_ and w_2_) of two sensory inputs (S_1_ and S_2_) onto the read-out neuron in EC_L2/3_ to simulate the ITDP observed in acute slices (a, c). f. Case 1: Slice simulation – HC-EC_L2/3_ is intact but EC_L2/3_-HC connections are severed due to plane of sectioning simulating the acute slice condition. Pairing HC feedback with S_1_ sensory input leads to potentiation of S_1_ and increased weight w_1_ with higher readout (behavioral output), while the unpaired S_2_ sensory input and its read-out remain unchanged. g. Case 2: Iterative HC-EC loop – Case 1 likely underestimates the effect due to severed axons (connections) between EC and HC. Upon simulating an intact EC_L2/3_-HC-EC_L2/3_ loop, pairing HC feedback with S_1_ sensory input leads to a higher potentiation of S_1_ than seen in case 1 and increased w_1_.

**Fig. 4.**
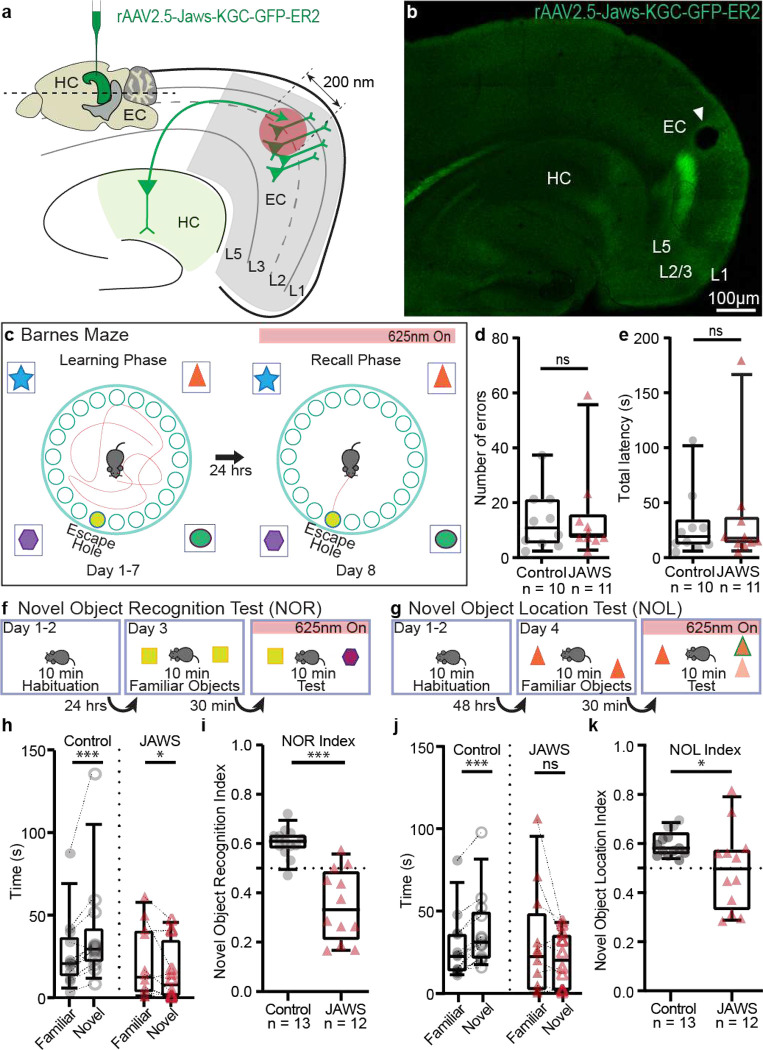
*in vivo* optogenetic silencing of the HC-EC_L2/3_ circuit impairs novelty detection in object recognition and object-place association tasks. a. Schema showing injection of recombinant AAV 2.5 JAWs-KGC-GFP-ER2 in the dorsal CA1 region of the Hippocampus (HC) and optical silencing of hippocampal projections (green arrow) expressing red-shifted halorhodopsin JAWS using red-light (680 nm) delivered through optical fibers (position marked by the red spot) of diameter 200 nm, placed over in entorhinal cortex layer 2/3 (EC_L2/3_). b. Representative confocal image of a horizontal brain slice from a mouse in the experimental cohort where JAWS-GFP was injected into dorsal CA1. GFP^+^ hippocampal axons can be seen in EC_L5_ and EC_L2/3_. The hole (white arrow head) in EC_L2/3_ marks the position of the optical fiber through which red-light was delivered to silence HC-EC_L2/3_ circuit. c. Experimental design of Barnes maze test. In the “learning” phase (day 1–7) animal is placed in the escape box for 1 min. Then the animal is placed at the center of the maze and allowed to freely explore the maze and its holes for 3 minutes or until it finds the escape hole (data not shown). In the “test” phase, animal is allowed to explore the maze until it finds the escape hole. During this test phase, laser light pulse of 625 nm is delivered to both control and JAWS injected animals. d. Comparison of the total number of errors made by the animal counted as the number of times it explores different holes including the escape hole without entering the escape box, in “test” phase. Box and whisker plots show the median number of errors made by the cohort of animals (control – black, JAWS – red), lower/upper quartile, 10–90^th^ percentiles. Each gray dot and red triangle represents the number of errors made by an individual control and JAWs animal respectively. Statistical significance tested with Mann Whitney test, p – value = 0.9835. e. Comparison of the total time for which the animal explored the arena (latency) before entering the escape box, in “test” phase. Box and whisker plots show the median latency of entering the escape box by the cohort of animals (control – black, JAWS – red), lower/upper quartile, 10–90^th^ percentiles. Each gray dot and red triangle represents the latency of an individual control and JAWs animal respectively. Statistical significance tested with Mann Whitney test, p – value = 0.9265. f. Experimental design of Novel Object Recognition (NOR) test. In the “habituation” phase (day 1–2) the animal is placed in an open field arena (without any objects) and allowed to freely explore the arena for 10 minutes each day. In the “test” day (day 3) the animal is placed in the arena with two identical objects. Animals are allowed to explore and familiarize with the objects for 10 minutes. After 30 minutes of rest in home cage, the animal is placed in the arena but one of the objects is replaced with a novel and different object. Animals are allowed to freely explore the two objects for 10 minutes. During this “test” phase, laser light pulse of 625 nm is delivered to both control and JAWS injected animals. g. Experimental design of Novel Object Location (NOL) test. In the ‘habituation’ phase (day 1–2) the animal is placed in an open field arena (without any objects) and allowed to freely explore the arena for 10 minutes each day. In the ‘test’ day (day 4) the animal is first placed in the arena with two identical objects. The animal is allowed to explore and familiarize itself with the objects for 10 minutes. 30 minutes after the animal is removed from the arena one of the two ‘familiar’ objects is moved to a new location in the arena. The animal is again allowed to explore the two object locations for ten minutes. During this test phase, laser light pulse of 625nm is delivered to both control and JAWS injected animals. h. Comparison of the time that the animals spent exploring the familiar vs novel objects during the test trial of the NOR task. Box and whisker plots show the median exploration time of the cohort of animals (control – black, JAWS – red), lower/upper quartile, 10–90^th^ percentiles. Each gray dot (solid – familiar object, open – novel object) and red triangle (solid – familiar object, open – novel object) represents the exploration time of an individual control and JAWs animal respectively. Statistical significance tested with Wilcoxon matched-pairs signed rank test for both cohorts, p – value = 0.0007 for control, p – value = 0.0425 for JAWs injected animals. i. Box and whisker plots showing the median novel object recognition index for the cohort of animals (control – black, JAWS – red), lower/upper quartile, 10–90^th^ percentiles during NOR test trial. Each gray dot and red triangle represents the NOR index an individual control and JAWs animal respectively. Statistical significance tested with unpaired t-test, p – value <0.0001. j. Comparison of the time that the animals spent exploring the familiar vs novel object locations during the test trial of the NOL task. Box and whisker plots show the median exploration time of the cohort of animals (control – black, JAWS – red), lower/upper quartile, 10–90^th^ percentiles. Each gray dot (solid – familiar object location, open – novel object location) and red triangle (solid – familiar object location, open – novel object location) represents the exploration time of an individual control and JAWs animal respectively. Statistical significance tested with Wilcoxon matched-pairs signed rank test for both cohorts, p – value = 0.0002 for control, p – value = 0.1726 for JAWs injected animals. k. Box and whisker plots showing the median novel object location index for the cohort of animals (control – black, JAWS – red), lower/upper quartile, 10–90^th^ percentiles during NOL test trial. Each gray dot and red triangle represents the NOL index an individual control and JAWs animal respectively. Statistical significance test with unpaired t-test, p – value = 0.0475.

## Data Availability

Experimental data from anatomy, physiology and behavior: All data, code, and materials used in the analysis and generation of figures will be made available on GitHUB (https://github.com/basulab-nyu/). Protocols for immunohistochemistry and behavioral experiments will be made available through the lab’s website (http://basulab.us). Morphological Reconstruction data: Following publication, neuronal morphological reconstruction data will be submitted to NeuroMorpho.Org. Computational Model Code: The models built for this study and the computer code will be uploaded online after publication on ModelDB (https://senselab.med.yale.edu/ModelDB/). Model Organism: All ‘model organisms’ generated by this project will be distributed freely and/or deposited into a repository/stock center making them available to the broader research community, either before or immediately after publication. In addition, we will provide relevant protocols and published genetic and phenotypic data upon request. Material transfers will be made with no more restrictive terms than in the Simple Letter Agreement (SLA) or the Uniform Biological Materials Transfer Agreement (UBMTA) and without reach through requirements. We will apply to have any transgenic mice generated during the project accepted by The Jackson Laboratory for distribution. If the mice are not accepted, we will breed and distribute the mice as needed. DNA and viral reagents: All virus construct plasmids will be submitted to Addgene repository and other commercial viral cores.
